# Phylogenetic Network Analysis Revealed the Occurrence of Horizontal Gene Transfer of 16S rRNA in the Genus *Enterobacter*

**DOI:** 10.3389/fmicb.2017.02225

**Published:** 2017-11-16

**Authors:** Mitsuharu Sato, Kentaro Miyazaki

**Affiliations:** ^1^Bioproduction Research Institute, Department of Life Science and Biotechnology, National Institute of Advanced Industrial Science and Technology, Tsukuba, Japan; ^2^Department of Computational Biology and Medical Sciences, Graduate School of Frontier Sciences, The University of Tokyo, Kashiwa, Japan

**Keywords:** ribosome, 16S ribosomal RNA, homogenization, horizontal gene transfer, phylogenetic network analysis, intragenomic recombination, intergenomic recombination, *Enterobacter*

## Abstract

Horizontal gene transfer (HGT) is a ubiquitous genetic event in bacterial evolution, but it seldom occurs for genes involved in highly complex supramolecules (or biosystems), which consist of many gene products. The ribosome is one such supramolecule, but several bacteria harbor dissimilar and/or chimeric 16S rRNAs in their genomes, suggesting the occurrence of HGT of this gene. However, we know little about whether the genes actually experience HGT and, if so, the frequency of such a transfer. This is primarily because the methods currently employed for phylogenetic analysis (e.g., neighbor-joining, maximum likelihood, and maximum parsimony) of 16S rRNA genes assume point mutation-driven tree-shape evolution as an evolutionary model, which is intrinsically inappropriate to decipher the evolutionary history for genes driven by recombination. To address this issue, we applied a phylogenetic network analysis, which has been used previously for detection of genetic recombination in homologous alleles, to the 16S rRNA gene. We focused on the genus *Enterobacter*, whose phylogenetic relationships inferred by multi-locus sequence alignment analysis and 16S rRNA sequences are incompatible. All 10 complete genomic sequences were retrieved from the NCBI database, in which 71 16S rRNA genes were included. Neighbor-joining analysis demonstrated that the genes residing in the same genomes clustered, indicating the occurrence of intragenomic recombination. However, as suggested by the low bootstrap values, evolutionary relationships between the clusters were uncertain. We then applied phylogenetic network analysis to representative sequences from each cluster. We found three ancestral 16S rRNA groups; the others were likely created through recursive recombination between the ancestors and chimeric descendants. Despite the large sequence changes caused by the recombination events, the RNA secondary structures were conserved. Successive intergenomic and intragenomic recombination thus shaped the evolution of 16S rRNA genes in the genus *Enterobacter*.

## Introduction

Based on the structural complexity of the ribosome (Brodersen et al., 2002; Klein et al., 2004; Schuwirth et al., 2005), it was believed that each ribosomal component is species-specific and the genes have seldom experienced HGT (Jain et al., 1999). In particular, the 16S rRNA gene has been recognized as the “ultimate chronometer” (Woese, 1987) and the sequence is widely used for taxonomic classification of bacteria (Lane et al., 1985; Woese, 1987; Woese et al., 1990).

One unique feature of rRNA genes is that the genomes of many bacteria contain several copies (Stoddard et al., 2015). For example, *Escherichia coli* contains 7 copies per genome and *Bacillus subtilis* contains 13 copies per genome. The 16S rRNA gene copies in the same genome are, in general, almost identical (>99.5% identity); this concerted evolution of 16S rRNA genes is due to gene conversion (or homogenization) within the genome (Hillis et al., 1991; Liao, 1999, 2000). In *E. coli*, the frequency of such intragenomic recombination between 16S rRNA genes is as high as 5 × 10^-9^ per generation (Hashimoto et al., 2003). Such a high sequence similarity among the copies of the 16S rRNA gene is also reported for bacterial genomes; bacterial species (genomes), which contain dissimilar (showing less than 98% sequence identity) 16S rRNA genes, are rare, counting for only 28 out of 2,143 genomes investigated (Tian et al., 2015).

**FIGURE 1:**
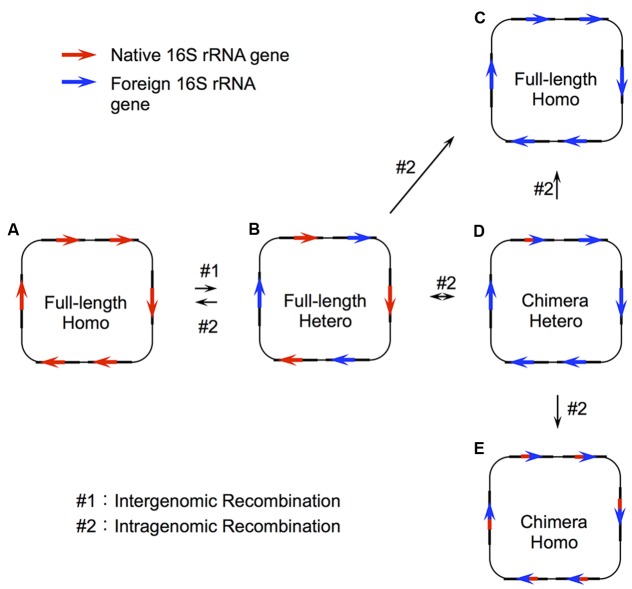
Two step recombination model for 16S rRNA genes. Recombination of the 16S rRNA gene takes place through two steps: intergenomic recombination (#1), followed by intragenomic recombination (#2). In the first step, a genetic fragment containing a foreign 16S rRNA gene is transferred to a host organism **(A)** to produce a genome with heterogeneous copies of the gene **(B)**. This initial step (#1) is the actual HGT (or interspecies exchange). From this heterogeneous status, there are four possible patterns of intragenomic recombination depending on the recombination sites. **(A)** All copies are taken over by the native 16S rRNA gene, **(C)** all copies are taken over by the full-length foreign 16S rRNA gene, and **(D)** part of the foreign 16S rRNA gene remains in some copy number. The status **(D)** would be changed to **(C)** or **(E)** through recursive intragenomic recombination.

Despite this general tendency of uniformity in 16S rRNA sequences within the same genomes, the genomes of several bacteria possess heterogeneous 16S rRNA genes (Mylvaganam and Dennis, 1992; Ueda et al., 1999; Yap et al., 1999; Acinas et al., 2004; Boucher et al., 2004) or chimeric 16S rRNA genes (Mylvaganam and Dennis, 1992; Eardly et al., 1996, 2005; Lan and Reeves, 1998; Ueda et al., 1999; Yap et al., 1999; Wang and Zhang, 2000; Schouls et al., 2003). With respect to the former, these instances can arise from inefficient intragenomic recombination after interspecies recombination (or HGT) [the evolutionary model for the 16S rRNA gene is illustrated in **Figure 1** (Schouls et al., 2003)] or each allele can play functionally different and essential roles for growth (Lopez-Lopez et al., 2007). With respect to the latter situation, chimeric sequences can arise through intergenomic recombination (or HGT, #1 in **Figure 1**) followed by intragenomic recombination between the copies in the same genome (Schouls et al., 2003) (#2 in **Figure 1**).

In addition to these natural instances, we recently demonstrated experimentally that the *E. coli* ribosome is capable of using foreign 16S rRNA from diverse bacterial origins with sequence identity as low as ∼78% (Kitahara et al., 2012; Miyazaki et al., 2017; Tsukuda et al., 2017). We also found that some domain- and helix-swapping were also permissible between distantly related species (Kitahara and Miyazaki, 2011; Tsukuda et al., 2017). Thus, as opposed to the “complexity hypothesis,” which claims that the major components included in complex supramolecules (or biosystems) do not transfer between species because such drastic genetic changes should disrupt the function of the whole molecule (or system) (Jain et al., 1999), 16S rRNA, which is the center of the 30S subunit and has many contacts with surrounding proteins, may be transferrable between species. Taken together, these instances suggest the occurrence of HGT in 16S rRNA genes, just as in enzyme-encoding genes (Kitahara and Miyazaki, 2013). The resulting products caused by HGT can be the replacement of the whole gene or segmental replacement depending on the recombination points (**Figure 1**).

Although several sporadic studies have reported HGT for 16S rRNA genes, little has been done to systematically investigate the possibility (and if present, the frequency) of HGT in 16S rRNAs. Recently, Tian et al. (2015) investigated 2,143 prokaryotic genomes, and they concluded that the frequency of HGT in 16S rRNA genes is “rare.” However, there are several significant mistakes in their study, primarily because of their use of an incorrect evolutionary model (**Figure 1**). Briefly, they focused their investigation on the genomes containing the 16S rRNA genes showing >2% dissimilarity (assuming that this dissimilarity could be brought by foreign bacterial 16S rRNA genes). However, such analysis overlooks the state E in **Figure 1**, and the state B when the initial dissimilar sequence is lost through intragenomic recombination where the degree of dissimilarity falls to a level below 2%. Another problem in their paper lies in their method used to detect recombination. Specifically, they used the phylogenetic tree approach based on maximum likelihood method, which is based on tree-shape evolution (driven by point mutations) and inadequate to decipher evolutionary history of genes including recombination.

To complement all these drawbacks, we here applied a “phylogenetic network” method (Bandelt, 1994), which has previously been used to detect evolutionary events, such as recombination (Kitano et al., 2009, 2012), gene conversion (Kitano and Saitou, 1999; Ezawa et al., 2010) and gene fusion (Tomiki and Saitou, 2004), in homologous alleles. For example, Kitano et al. reported that genetic recombination created the haplogroups of the ABO blood type genes of gibbon monkeys (Kitano et al., 2009) and humans (Kitano et al., 2012). We targeted the genus *Enterobacter*, because the phylogenetic relationships inferred by multi-locus sequence alignment (MLSA) analysis and 16S rRNA-based analysis in this genus are incompatible (Brady et al., 2013). We collected all 10 complete genomic sequences of *Enterobacter* from the NCBI database and made phylogenetic analysis for 16S rRNA genes therein and MLSA analysis for the genomes. The results suggested a topological difference between the phylogenetic trees, confirming the results by Brady et al. (2013) and the occurrence of recombination-driven evolution in 16S rRNA genes. We then applied phylogenetic network analysis to the 16S rRNA genes, which suggested that there were three ancestral groups of 16S rRNA genes among the 10 groups, of which two groups appeared to create a further seven groups, through five recombination events. The present results strongly suggest that HGT is a major driving force for the evolution of 16S rRNA genes in the genus *Enterobacter*.

## Materials and Methods

### Dataset Collection

16S rRNA genes of *Enterobacter* were retrieved from the National Center for Biotechnology Information (NCBI) database on July 14, 2015. The information (name of each sequence, genome accession numbers, locations in the genome and species name) of the 71 retrieved *Enterobacter* sequences is shown in Supplementary Table S1.

### Multiple Sequence Alignment

Multiple sequence alignment of 16S rRNA genes was carried out using MUSCLE (Edgar, 2004) implemented in the MEGA 6.0 software (Tamura et al., 2013) with default parameters. All single insertion sites of *Enterobacter* sequences were eliminated after multiple sequence alignment. Singleton sites and PISs were identified using MEGA 6.0 software (Tamura et al., 2013). *E. coli* site numbering was used.

### Phylogenetic Tree Analysis

A phylogenetic tree of 16S rRNA genes was reconstructed using the neighbor-joining method (Saitou and Nei, 1987) implemented in the MEGA 6.0 software (Tamura et al., 2013) with the Kimura two-parameter distance (Kimura, 1980). Bootstrap values were calculated with 1,000 resamples. A parsimonious phylogenetic network was constructed following the procedures set out in Bandelt (1994) and Saitou and Yamamoto (1997).

### Determination of Non-randomness of the Order of PISs

A two-sample runs test (Takahata, 1994; Ezawa et al., 2006) was employed to investigate the random/non-random distribution of PISs in the 16S rRNA sequences. Detailed calculation procedures are described in the Supplementary Information. The results were benchmarked following the procedure by Takahata (1994).

### MLSA Analysis

Multi-locus sequence alignment analysis was carried out for the 10 representative *Enterobacter* genomes using the following procedure. (1) Retrieved 609 essential genes, which are listed in the Database of Essential Genes (Zhang and Lin, 2009), from the *E. coli* MG1655 genome (NC_000913.3). (2) Identified 194 essential genes (in the 609 genes as described above), which are shared by selected *Enterobacter* genomes. (3) Each gene was translated into their amino acid sequence, which were multiply aligned using MUSCLE 3.8.31 (Edgar, 2004). (4) Removed poorly aligned regions and divergent regions using Gblocks 0.91 (Castresana, 2000). When more than 20 continuous amino acid sites were found to be different, these sites were also manually removed. (5) Concatenated all the trimmed amino acid sequences. (6) Constructed a neighbor-joining tree using MEGA. 6.0 software with the JTT matrix-based method (Jones et al., 1992) with 1,000 bootstrap resampling.

## Results and Discussion

### Data Collection

We retrieved 10 complete genomic sequences of *Enterobacter* from the National Center for Biotechnology Information (NCBI) database, which were designated G1 to G10 (Supplementary Table S1). Among them, all but one genome (G5) contained multiple 16S rRNA gene copies. G1, G3, and G5 contained seven copies; G2, G4, G6, G7, and G8 contained eight copies, and G10 contained nine copies (Supplementary Table S1). Overall, a total of 71 16S rRNA genes was identified in the 10 genomes.

### Intragenomic Uniformity of 16S rRNA Gene Copies

**FIGURE 2:**
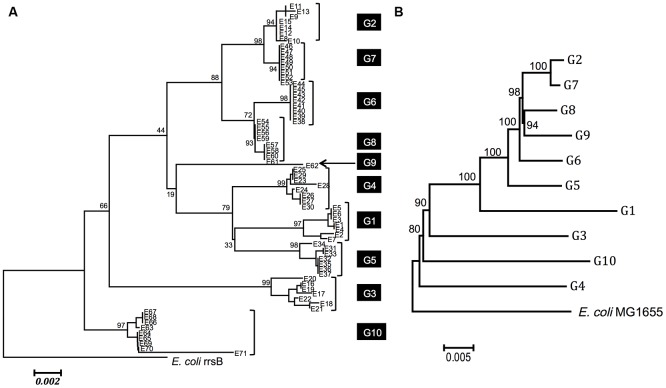
Phylogenetic relationships among *Enterobacter* species. **(A)** Phylogenetic relationship based on 16S rRNA sequences. A phylogeny was reconstructed using the neighbor-joining method (Saitou and Nei, 1987) with the Kimura two-parameter distance (Tamura et al., 2013). Seventy-one *Enterobacter* sequences from 10 genomes (G1 to G10) are shown. All 16S rRNA gene copies from each genome are clustered. The strains of each genome are as follows: G1: *Enterobacter* sp. 638 (NC_009436.1), G2: *Enterobacter cloacae* subsp. *cloacae* ATCC 13047 (NC_014121.1), G3: *E. cloacae* SCF1 (NC_014618.1), G4: *E. aerogenes* KCTC 2190 (NC_015663.1), G5: *E. asburiae* LF7a (NC_015968.1), G6: *E. cloacae* EcWSU1 (NC_016514.1), G7: *E. cloacae* subsp. *dissolvens* SDM (NC_018079.1), G8: *E. cloacae* subsp. *cloacae* ENHKU01 (NC_018405.1), G9: *E. cloacae* subsp. *cloacae* NCTC 9394 (NC_021046.1) and G10: *E.* sp. R4-368 (NC_012500.1). The numbers near the nodes are bootstrap values (1,000 resamplings). **(B)** Phylogenetic relationship based on MLSA analysis. MLSA tree for 10 *Enterobacter* genomes (G1–G10) and the *E. coli* genome based on 194 concatenated amino acid sequences. A phylogeny was reconstructed using the neighbor-joining method (Saitou and Nei, 1987) with the JTT matrix-based method (Jones et al., 1992). The amino acid sites including gaps and missing data were eliminated. In total, the protein-coding genes contained 61,774 amino acid positions. The 1,000 resampling bootstrap values are shown. The phylogenetic relationships among these genomes are strongly supported by high bootstrap value (>80).

**Figure 2A** shows a neighbor-joining tree of the 71 *Enterobacter* 16S rRNA gene sequences with *E. coli* MG1655 *rrsB* as an outgroup. All of the 16S rRNA genes that originated from the same genome shared high sequence identities (≥99%) and clustered with high bootstrap values (≥93). This indicates the occurrence of intragenomic recombination (homogenization) between the copies (Hillis et al., 1991; Liao, 1999, 2000). In contrast, bootstrap values for each node were, in general, low, suggesting uncertain topology between the genomes.

To investigate the phylogenetic relationships of 16S rRNA gene sequences between clusters (genomes), we selected representative 16S rRNA genes from each cluster. As a rule, we selected one ancestral sequence (closest to the outgroup [*E. coli*]) and one sequence from each cluster to cover the variety. For example, for G1, E7 branches from the ancestral node and E1, E3, E4, E5, and E6 clustered (Supplementary Figure S1A). Thus, E1 and E7 were selected as the representatives of G1. Similarly, from each genome, we selected E8 and E9 from G2 (Supplementary Figure S1B), E19, E20, and E22 from G3 (Supplementary Figure S1C), E23 and E26 from G4 (Supplementary Figure S1D), E32 and E33 from G5 (Supplementary Figure S1E), E54 and E57 from G8 (Supplementary Figure S1F) and E63, E64, and E66 from G10 (Supplementary Figure S1G). For G6 and G7, we selected one sequence because all copies in the genomes were identical in these strains (E46 and E38, respectively). For G9, only a single 16S rRNA copy (E62) was used in the analysis. In total, we selected 19 representative sequences from 10 *Enterobacter* genomes (indicated by dots in Supplementary Figure S1).

### MLSA Analysis

Brady et al. (2013) have reported that the phylogenetic relationships inferred by MLSA analysis using four protein-coding essential genes (*rpoB*, *gyrB*, *infB*, and *atpD*) and 16S rRNA-based analysis were incompatible. We performed a similar analysis for the 10 selected genomes (G1–G10) using a more comprehensive set of essential genes. We first retrieved 609 genes that were known to be essential in *E. coli*, from which 194 genes were selected. These genes were present in all 10 genomes. The 194 protein-coding genes (61,774 amino acids total) were as follows; *accA, accB, accC, aceF, ackA, acpP, adk, alaS, argS, aroK, asnS, aspS, atpC, atpF, can, csrA, cvpA, cydA, cydB, cysS, dadA, dapF, der, dnaA, dnaB, dnaE, dnaG, dnaK, dxs, eno, era, fabA, fabB, fabG, fabI, fbaA, ffh, fis, folA, folD, frr, ftsA, ftsH, ftsI, ftsQ, ftsY, ftsZ, fusA, gapA, glmS, glnS, glyA, glyQ, glyS, gmk, gpsA, groL, grpE, gshB, gyrA, gyrB, hemE, hfq, hisS, hns, holC, ileS, infA, infB, infC, iscS, ispB, ispE, ispH, ispU, lepB, leuS, lolC, lpxA, lpxC, lpxD, metG, metK, miaA, minD, mraY, mreB, mukB, mukF, murC, murE, murI, nrdA, nrdB, nusG, obgE, parE, pgk, pheS, pheT, plsB, pnp, ppiB, priB, proS, purB, pyrG, ribA, ribB, ribE, ribF, rnc, rne, rnpA, rplB, rplC, rplD, rplE, rplF, rplJ, rplL, rplM, rplN, rplO, rplP, rplQ, rplR, rplS, rplT, rplU, rplV, rplW, rplX, rplY, rpmA, rpmB, rpmC, rpmD, rpmH, rpoA, rpoB, rpoC, rpoD, rpoE, rpoH, rpsA, rpsB, rpsC, rpsD, rpsE, rpsF, rpsG, rpsH, rpsI, rpsJ, rpsK, rpsL, rpsM, rpsN, rpsO, rpsP, rpsQ, rpsR, rpsS, rpsT, secA, secD, secE, secY, serS, slyD, spoT, ssb, sucA, sucB, suhB, thrS, tktA, topA, trmD, trpS, tsf, tufA, tufB, tyrS, valS, ybeY, ybgC, yceQ, ychF, yidC, yqgE, yqiB, zipA*. The deduced amino acid sequences for these genes were trimmed, concatenated, and used for phylogenetic analysis. **Figure 2B** shows the phylogenetic relationship between these genomes, whose topology was different from that constructed based on the 16S rRNA (**Figure 2A**), confirming the notion by Brady et al. (2013).

### Multiple Occurrences of HGT in 16S rRNA Genes in *Enterobacter*

**FIGURE 3:**
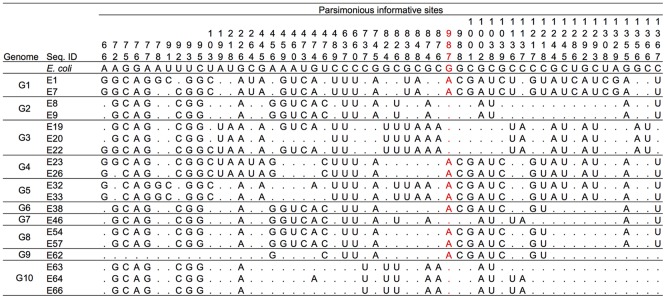
Multiple alignment of 54 parsimonious informative sites (PISs) of 19 representatives. Dots indicate nucleotides identical to those in the *E. coli* sequence. The PIS numbers correspond to the nucleotide positions in the 16S rRNA gene of *E. coli*.

**FIGURE 4:**
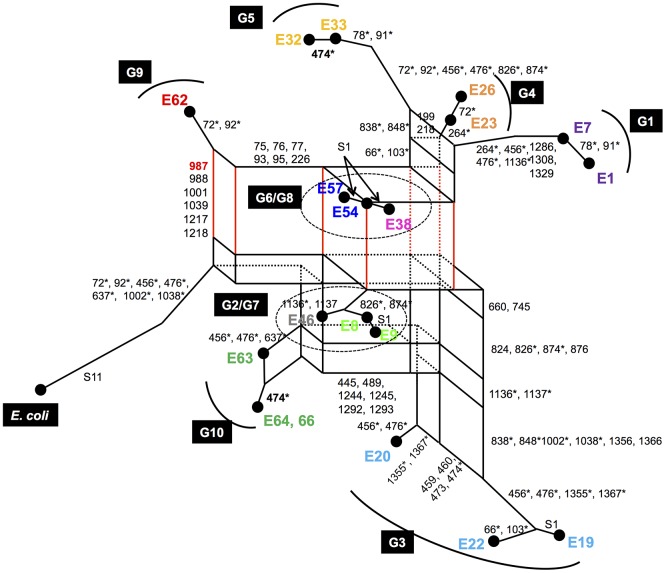
A parsimonious phylogenetic network of 19 representative sequences from 10 *Enterobacter* genomes. The sequences were selected from the 10 complete genomes as described in the text. Sequences derived from the same genome are shown in the same color. The numbers near each side are the PISs responsible for those sides. The PISs with asterisks are responsible for the sides, which were not drawn in three dimensions. Numbers prefixed with “S” are numbers of singletons.

In the multiple alignments of 20 16S rRNA gene sequences (19 representative sequences from *Enterobacter* plus *E. coli*), there were a total of 82 variable sites. Of these sites, 15 were singletons and 67 were PISs. Among the 67 PISs, those carrying more than three nucleotides (11 PISs) were removed from the analysis. Two PISs that contained gaps were also removed from the analysis. Overall, 15 singletons and 54 PISs were used for phylogenetic network analysis. A multiple alignment of the 54 PISs of the 19 representatives is shown in **Figure 3**. Based on this PIS alignment and 15 singleton sites, a parsimonious phylogenetic network was reconstructed, following the procedure as described previously (Bandelt, 1994; Saitou and Yamamoto, 1997) (**Figure 4**). The numbers along each side indicate the corresponding PISs that separate one group from another. For example, *E. coli*, G2/G7 (E8, E9, E46), G3 (E19, E20, E22) and G10 (E63, E64, E66) have the nucleotide ‘G’ at PIS 987 (red-colored numbers in **Figures 3**, **4**), whereas others (G1 [E1, E7], G4 [E23, E26], G5 [E32, E33], G6/G8 [E38, E54, E57] and G9 [E62]) have ‘A’ at the same position. Thus, the genomic groups are separated by PIS 987. Similarly, in *E. coli*, G2/G7, G3 and G10 are separated from G1, G4, G5, G6/G8, and G9 by PISs 988, 1001, 1039, 1217, and 1218. These PISs are located on the same vertical side with PIS 987.

In a parsimonious phylogenetic network, recombination results in the formation of reticular structures. In the present case, there are many reticules (or vertical and horizontal sides) in the parsimonious phylogenetic network, suggesting multiple occurrences of recombination (Kitano et al., 2009, 2012). From the reticule, the following evolutionary information can be obtained (Kitano et al., 2012; Saitou and Kitano, 2013):

(1) The recombinant sequence is located at the corner opposite the outgroup.
(2) The parental sequences are located at the corner of another diagonal of the same reticule.
(3) The two sides (vertical and horizontal) of the reticule are dominated by the upstream or downstream PISs in question.

To deduce each recombination event in **Figure 4**, we dissected the whole network into several groups and attempted to identify hypothetical recombinants and their parents in each group. Let us focus on the reticule consisting of G2/G7, G6/G8, G9, and *E. coli* as an example (Supplementary Figure S2A). In this case, G6/G8 are the hypothetical recombinants because they are located in the corner opposite the outgroup (*E. coli*), and G2/G7 and G9 are the hypothetical parents, because they are located in the two corners of another diagonal (Supplementary Figure S2A). The PISs of one side (side 1, colored in red) of this reticule are 75, 76, 77, 93, 95, 226, 459, 460, 473, 474, 1,355, and 1,367, and the PISs of another side (side 2, colored in blue) are 987, 988, 1,001, 1,039, 1,217, and 1,218 (Supplementary Figure S2A). The arrangement of PISs on sides 1 and 2 is as follows (the PISs from side 2 are shown in bold typeface):

75, 76, 77, 93, 95, 226, 459, 460, 473, 474, **987, 988, 1,001, 1,039, 1,217, 1,218**, 1,355, 1,367.

This consecutive distribution of the PISs strongly suggests that the G2/G7 group and G9 recombinantly created the G6/G8 group, and the recombination points are located between PISs 474 and 987, and 1,218 and 1,355, suggesting the occurrence of two recombinations.

To confirm the non-random distribution of these PISs statistically, we employed the two-sample runs test (Takahata, 1994; Ezawa et al., 2006). A “run” is defined as a sequence of letters of the same kind bounded by letters of another kind, with the exception of the first and last positions (Saitou and Yamamoto, 1997). In this case, the total number of PISs is 20 (*n* = 20), of which 14 are derived from side 1 (α = 12) and 6 from side 2 (β = 6). The sequence is separated into three blocks (PISs 72–474, 987–1,218, and 1,355–1,367), so that the number of runs (Z) is three. The probability of the number of runs becoming fewer than three is significantly low (*P* = 0.0005, < 0.05), indicating that the PISs are distributed non-randomly. The details of the calculation for each side are described in the Supporting Information.

### Recombination Is a Major Driving Force for the Evolution of *Enterobacter* 16S rRNA Genes

**FIGURE 5:**
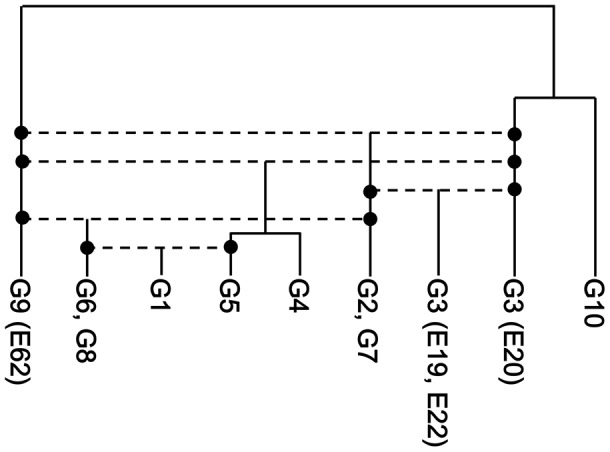
The recombination history of the 16S rRNA gene of *Enterobacter*. The five inferred recombination events are indicated by horizontal dotted lines. The phylogenetic relationships of the non-recombinants (E20, G10, and G9) were inferred using the neighbor-joining method (Saitou and Nei, 1987) with the Kimura two-parameter distance (Kimura, 1980).

Similar analysis was performed for the other groups in the network. We found that G1, G2/G7, G4/G5 and two of G3 (E19 and E22) were the recombinants, whose hypothetical parents are G5 and G6/G8, G3 (E20) and G9, G3 (E20) and G9 and G3 (E20) and G2/G7, respectively (Supplementary Figures S2B–E). The results of the analysis are summarized in Supplementary Table S2 (see also Supporting Information) and the evolutionary history of representative *Enterobacter* 16S rRNA genes is illustrated in **Figure 5**, in which horizontal dotted lines indicate recombination events. All 16S rRNA gene copies of G1, G2, G3, G4, G6, G7, and G8, and six of seven 16S rRNA gene copies of G9 were created by intergenomic recombination between two parents, E62 (G9) and E20 (G3). G10 shares a lineage with E20, but did not serve as a parent. Collectively, a total of five intergenomic recombination events took place in *Enterobacter*.

As illustrated in **Figure 2A**, the majority of 16S rRNA gene copies in the same genome are highly homologous, which suggests that the existence of non-homogeneous 16S rRNA genes in the same genome is not favorable and intragenomic recombination (homogenization) is a “fast” process. In fact, the frequency of intragenomic recombination in *E. coli* 16S rRNA gene copies was estimated to be 5 × 10^-9^ per generation (Hashimoto et al., 2003), whereas the spontaneous point mutation rate is estimated to be 2.2 × 10^-10^ per generation per site in the same organism (Lee et al., 2012). Thus, the “transient states” would not be frequently found in the 16S rRNA genes in *Enterobacter*, but as a rare exception we identified a possible “on-going” homogenizing process in G9. In this genome, one copy (E20) was a parent and the others (E19 and E22) were recombinants between E20 and the G2/G7 group (**Figure 4**). Thus, E20 likely pre-existed in the genome and the second parent G2/G7 integrated into the cell (either in-genome or ex-genome), followed by recombination between the alleles. Many types of chimerization might have occurred, but subsequent intragenomic recombination should have selected one of those (i.e., E19/E22 type). We speculate that the relic E20 would eventually be replaced by the E19/E22 type.

### Recombination Does Not Perturb RNA Secondary Structures

**FIGURE 6:**
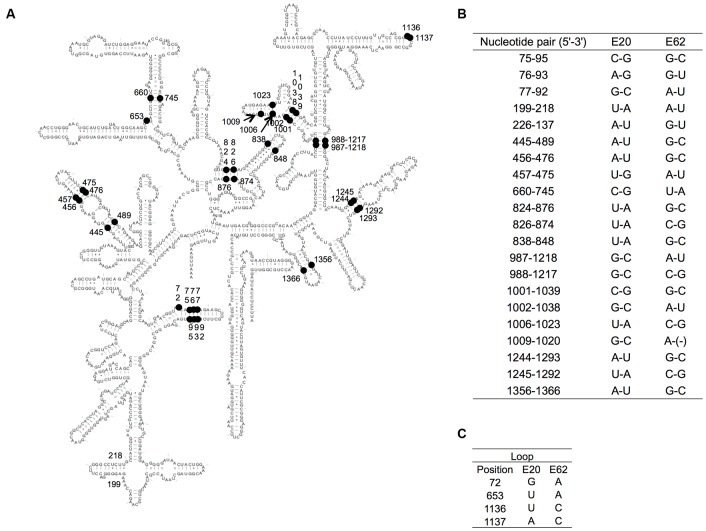
Variable nucleotides between E20 and E62. **(A)** The variable nucleotide positions between E20 and E62 of the secondary structure of 16S rRNA are indicated by black nodes. **(B)** The co-paired variable positions. **(C)** The variable positions in the loop. *E. coli* numbering.

In general, secondary structures are key for the functionality of 16S rRNAs and must be conserved during evolution (Laios et al., 2004). From this point of view, we mapped the different sites between two parental *Enterobacter* 16S rRNAs, E20 (of G3) and E62 (of G9) (**Figure 6A**). There were 46 different nucleotides, of which 42 were positioned in stem regions, which co-varied to maintain the base pairs in each recombinant (**Figure 6B**). With a sole exception, the 1,020th nucleotide (in h33) was C in E20 and missing (gap) in E62, which was found only in E20 in 19 representatives. Four nucleotides were located in loop regions (72, 653, 1,136, and 1,137) (**Figure 6C**). Our previous study suggests that all of these nucleotides are variable (72R, 653N, 1136N, 1137H) in *E. coli* 16S rRNA (Kitahara et al., 2012), suggesting them to be functionally neutral.

### Notes on Previous Studies

With the rapid increase in the number of bacterial genomic sequences, attempts have been made to investigate the occurrence of HGT in 16S rRNA genes. Tian et al. (2015) focused on the complete prokaryotic genomes and concluded that HGT is a rare event (Tian et al., 2015). More specifically, they first retrieved 2,143 complete prokaryotic genomes. They then selected 28 genomes that showed the pairwise identities between 16S rRNA genes that were less than 98%. They assumed that such “dissimilar” 16S rRNA genes could be brought by HGT from different bacterial species. Next, to investigate the origin of the dissimilarity, they used each 16S rRNA gene sequence in the 28 genomes as a query and BLAST searched for the top 50 hits in the NCBI database. They then reconstructed a maximum likelihood tree of these hits and queries. When the query sequence positioned in a cluster different from the host lineage, they considered that the query gene was brought by HGT. For example, they found one of the two copies of the 16S rRNA gene (copy A) of strain *Chlamydia trachomatis* clustered with *Chlamydia trachomatis* sequences, and the other (copy B, 97.1% identical to copy A) clustered with *C. suis* sequences with only one mismatch over 1,520 bases. Based on this, they concluded that the copy B was horizontally transferred from *C. suis* to the *C. trachomatis* genome (Tian et al., 2015).

However, their analysis (and thus the deduced results) suffers from several fatal errors. First, their analysis focused only on some specific states of the whole HGT process (states B and D in **Figure 1**). A high rate of intragenomic recombination is already known (Hashimoto et al., 2003) and their analysis simply approved the results. Additionally, some argue that all copies of the 16S rRNA sequences are chimeric (Schouls et al., 2003), and that they originated by HGT followed by intragenomic recombination (state E in **Figure 1**). However, Tian’s criteria (i.e., 2% dissimilarity) could overlook such chimeric sequences. In this study, we also found that, out of the 10 genomes, 8 *Enterobacter* genomes contained chimeric 16S rRNA gene copies that were nearly identical (<0.4% dissimilarity). The 28 genomes that Tian et al. (2015) identified may be the transition state of intragenomic recombination, which is certainly rare as suggested by previous studies (Hashimoto et al., 2003).

The analytical procedure used after the selection of the 28 genomes is also problematic, in that they used Blast search (using individual 16S rRNA gene sequences as a query) to identify genomes. However, in the case of segmental HGT (which usually results in the creation of chimeric genes), the segmental sequence will not be recovered from the database due its short length.

Furthermore, the phylogenetic tree method is inadequate for the detection of recombination as it is beyond the application limit as described above. Overall, their analysis will underestimate the frequency of HGT.

## Conclusion

We identified at least five intergenomic recombination events within the *Enterobacter* genome. Taking the high susceptibility of 16S rRNAs for point mutations, which can disrupt secondary structure (Laios et al., 2004), into account, it is reasonable to think that the evolution of 16S rRNA genes was facilitated by HGT. Recombination-based evolution (intergenomic, followed by intragenomic) effectively generated a higher order of sequence diversity, shaping a specific phylogenetic group, the genus *Enterobacter*.

In this study, we investigated the possibility (and frequency) of HGT in 16S rRNA genes using a bioinformatic approach. Very recently, through an experimental approach, we found an unexpectedly high functional compatibility between the 16S rRNA of *E. coli* and Acidobacteria, which are different at the phylum level (Tsukuda et al., 2017). Based on the high compatibility of 16S rRNAs, we proposed a new evolutionary model for 16S rRNA (and the ribosome). The model, which we named the “Cradle Model,” suggests that bacterial 16S rRNAs are significantly connected by a neutral network, and thus, functional interactions can be maintained before and after interspecies exchange of 16S rRNA. Taken together, our results strongly suggest that 16S rRNA genes are amenable to HGT.

## Author Contributions

MS and KM designed the study, MS conducted the data analysis and MS and KM wrote the manuscript.

## Conflict of Interest Statement

The authors declare that the research was conducted in the absence of any commercial or financial relationships that could be construed as a potential conflict of interest.

## Abbreviations

HGT: horizontal gene transfer
PIS: parsimonious informative site
