# Supplementary material for: Phylogenetic Network Analysis Revealed the Occurrence of Horizontal Gene Transfer of 16S rRNA in the Genus Enterobacter
Source: Front Microbiol. 2017 Nov 16;8:2225. doi: 10.3389/fmicb.2017.02225 (PMC5688380; doi:10.3389/fmicb.2017.02225)
Supplement: Supplementary file 1 [file Table_1.PDF]

Table 1 List of 16S rRNA sequences in the genus *Enterobacter* retrieved from the NCBI database

Genome ID (accession)	Sequence ID	Genome locus	Strain
NC_000913.3	*E. coli*	4166659-4168200	*Escherichia coli* str. K-12 substr. MG1655
G1 (NC_009436.1)	E1	250882-252426	*Enterobacter sp.* 638
	E2	838233-839777	
	E3	3346252-3347796	
	E4	4027485-4029029	
	E5	4273048-4274592	
	E6	4364121-4365665	
	E7	4469715-4471259	
G2 (NC_014121.1)	E8	1015758-1017300	*Enterobacter cloacae* subsp. cloacae ATCC13047
	E9	265437-266982	
	E10	380924-382468	
	E11	4022288-4023830	
	E12	4788527-4790069	
	E13	5066249-5067791	
	E14	5152784-5154334	
	E15	5269663-5271205	
G3 (NC_014618.1)	E16	448966-450494	*Enterobacter cloacae* SCF1
	E17	1199254-1200781	
	E18	3787696-3789223	
	E19	4480465-4481992	
	E20	4525723-4527249	
	E21	4624500-4626027	
	E22	4758193-4759721	
G4 (NC_015663.1)	E23	220089-221642	*Enterobacter aerogenes* KCTC_2190
	E24	996110-997664	
	E25	1512350-1513903	
	E26	1604515-1606068	
	E27	1693658-1695211	

	E28	1738693-1740246	
	E29	2128391-2129944	
	E30	2536976-2538529	
	E31	257230-258757	
	E32	865115-866642	
	E33	3542582-3544109	
G5 (NC_015968.1)	E34	4285323-4286850	*Enterobacter asburiae* LF7a
	E35	4531152-4532679	
	E36	4624193-4625719	
	E37	4749056-4750583	

G6 (NC_016514.1)	E38	237096-238638	*Enterobacter cloacae* EcWSU1
	E39	354481-356023	
	E40	855718-857260	
	E41	3551486-3553028	
	E42	4249983-4251525	
	E43	4492055-4493597	
	E44	4578459-4580001	
	E45	4688126-4689668	
G7 (NC_018079.1)	E46	277104-278653	*Enterobacter cloacae* subsp. dissolvens_SDM
	E47	385086-386635	
	E48	923783-925332	
	E49	3712952-3714501	
	E50	4459451-4461000	
	E51	4729483-4731032	
	E52	4815991-4817540	
	E53	4921505-4923054	
G8 (NC_018405.1)	E54	269132-270685	*Enterobacter cloacae* subsp. cloacae ENHKU01
	E55	402398-403951	
	E56	967745-969298	
	E57	3598483-3600037	
	E58	4298149-4299703	
	E59	4501110-4502663	
	E60	4596907-4598461	
	E61	4724725-4726279	
G9 (NC_021046.1)	E62	119494-121022	*Enterobacter cloacae* subsp. cloacae_NCTC_9394
G10 (NC_021500.1)	E63	1700903-1702442	*Enterobacter sp.* R4-368
	E64	2537017-2538556	
	E65	3030101-3031640	
	E66	3155371-3156910	
	E67	3161615-3163154	

E68	3250119-3251658
E69	3294413-3295952
E70	3995107-3996646
E71	4001483-4002948
