# Supplementary material for: Phylogenetic Network Analysis Revealed the Occurrence of Horizontal Gene Transfer of 16S rRNA in the Genus Enterobacter
Source: Front Microbiol. 2017 Nov 16;8:2225. doi: 10.3389/fmicb.2017.02225 (PMC5688380; doi:10.3389/fmicb.2017.02225)
Supplement: Supplementary file 2 [file Table_2.PDF]

Table 2 List of the detected recombination and the results of runs test.

Recombinant	Parent 1	Parent 2	Runs test	Network
G1 (E1, E7)	G5 (E32, E33)	G6 and G8 (E38, E54, E57)	P=0.0088	Fig. S2B
G2/G7 (E8, E9, E46)	G9 (E62)	G3 (E20)	P=0.028	Fig. S2E
G4/G5 (E23, E26, E32, E33)	G9 (E62)	G3 (E20)	P=0.0002	Fig. S2C
G3 (E19, E22)	G3 (E20)	G2 and G7 (E8, E9, E46)	P=0.0023	Fig. S2D
G6/G8 (E38, E54, E57)	G9 (E62)	G2 and G7 (E8, E9, E46)	P=0.001	Fig. S2A
