# Supplementary material for: Phylogenetic Network Analysis Revealed the Occurrence of Horizontal Gene Transfer of 16S rRNA in the Genus Enterobacter
Source: Front Microbiol. 2017 Nov 16;8:2225. doi: 10.3389/fmicb.2017.02225 (PMC5688380; doi:10.3389/fmicb.2017.02225)
Supplement: Supplementary file 3 [file Data_Sheet_1.PDF]

Supplementary Information

Two-sample Runs Test

To determine the randomness of the order of PISs, we used the two-sample runs test (Takahata 1994; Ezawa et al., 2006). This test calculates the probability that the number of 'runs' is fewer than that observed. A 'run' is a sequence of the same letter bounded by different letters. For example, the sequence 'aaabbbaabb' has four runs (aaa, bbb, aa and bb). The probability of observing a particular number of runs (Z) was calculated by the α (number of 'a' in the example case) and β (number of 'b' in the example case) values:

$$\mathrm{P}\{Z = 2k\} = 2\binom{\alpha - 1}{k - 1}\binom{\beta - 1}{k - 1}\binom{n}{\alpha}^{-1}$$

when $Z = 2k$ is even, and

$$\mathrm{P}\{Z = 2k + 1\} = \left\{\binom{\alpha - 1}{k}\binom{\beta - 1}{k - 1} + \binom{\alpha - 1}{k - 1}\binom{\beta - 1}{k}\right\}\binom{n}{\alpha}^{-1}$$

when $Z = 2k+1$ is odd. When both α and β exceed 10, the distribution of Z is approximately normal with mean (m) and variance (v):

$m = \frac{2\alpha\beta}{n} + 1$ and $v = \frac{2\alpha\beta(2\alpha\beta - n)}{n^2 - (n-1)}$

Therefore, if we transform Z into $\mathrm{N} = (Z - m)\sqrt{v}$, the distribution of N is normal with mean 0 and variance 1. Recombination is inferred when the observed number of runs is significantly small.

By applying the above equation, we determined the non-randomness of the order of PISs from the two sides of each reticule (**Supplementary Fig. 2)**. Details of the calculations follow.

Hypothetical Recombinant: G6/G8 (Supplementary Fig. 2A)

Because G6/G8 is located in the corner opposite the outgroup and G9 and G2/G7 are located in the two corners of another diagonal, G6/G8 is a hypothetical recombinant and G9 and G2/G7 are the hypothetical parents. Side 1 comprises 12 PISs (colored red), whereas side 2 comprises 6 PISs (colored blue). The order of PISs is as follows (PISs derived from side 2 are shown in bold typeface):

75, 76, 77, 93, 95, 226, 459, 460, 473, 474, **987, 988, 1001, 1039, 1217, 1218,** 1355, 1367

The number of runs (*Z*) is 3 (75-474, 987-1218 and 1355-1367), the number of PISs derived from side 1 (α) is 12 and the number of PISs derived from side 2 (β) is 6. The total number of PISs (n) is 18. Therefore, the sum of probability of the number of runs (*Z*) was calculated to be 2 and 3. When $Z = 2$:

$$\mathrm{P}\{Z = 2k\} = 2\binom{11}{0}\binom{5}{0}\binom{18}{12}^{-1} = 0.001$$

and when $Z = 3$:

$$\mathrm{P}\{Z = 2k + 1\} = \left\{\binom{11}{1}\binom{5}{0} + \binom{11}{0}\binom{5}{1}\right\}\binom{18}{12}^{-1} = 0.0009$$

The sum of these probabilities is 0.0001, which is significant at the 0.05% level.

Hypothetical recombinant: G1 (Supplementary Fig. 2B)

Because G1 is located in the corner opposite the outgroup and G5 and G6/G8 are located in both corners of another diagonal, G1 is a hypothetical recombinant and G5 and G6/G8 are the hypothetical parents. Side 1 comprises 10 PISs (colored red), whereas side 2 comprises 4 PISs (colored blue). The order of PISs is as follows (PISs from side 2 are shown in bold typeface):

66, 103, 445, **459, 460, 473, 474,** 489, 838, 848, 1244, 1245, 1292, 1293

The number of runs (*Z*) is 3 (66-445, 459-474 and 489-1293), the number of PISs derived from side 1 (α) is 10 and the number of PISs derived from side 2 (β) is 4. The total number of

PISs (n) is 14. Therefore, the sum of the probability of the number of runs (*Z*) was calculated to be 2 and 3. When $Z = 2$:

$$\mathrm{P}\{Z = 2k\} = 2\binom{9}{0}\binom{3}{0}\binom{14}{10}^{-1} = 0.002$$

and when $Z = 3$:

$$\mathrm{P}\{Z = 2k+1\} = \left\{\binom{9}{1}\binom{3}{0} + \binom{9}{0}\binom{3}{1}\right\}\binom{14}{10}^{-1} = 0.012$$

The sum of these probabilities is 0.014, which is significant at the 5% level.

Hypothetical recombinant: G2/G7 (Supplementary Fig. 2C)

Because G2/G7 is located in the corner opposite the outgroup and G3 (E20) and G9 are located in the two corners of another diagonal, G2/G7 is a hypothetical recombinant and G3 (E20) and G9 are the hypothetical parents. Side 1 comprises eight PISs (colored red), whereas side 6 comprises four PISs (colored blue). Note that the phylogenetic pattern of PISs 826 and 874 is the same as side 1, whereas the phylogenetic pattern of PISs 456, 476, 1002 and 1038 is the same as side 2. The order of PISs is as follows (PISs derived from side 2 are shown in bold typeface):

75, 76, 77, 93, 95, 226, **456, 476, 660, 745,** 826, 874, **1002, 1038**

The number of runs (*Z*) is four (75-226, 456-745, 826-874 and 1002-1038), the number of PISs originating from side 1 (α) is eight and the number of PISs originating from side 2 (β) is six. The total number of PISs (n) is 14. Therefore, the sum of the probability of the number of runs (*Z*) was calculated to be 2, 3 and 4. When $Z = 2$:

$$\mathrm{P}\{Z = 2k\} = 2\binom{7}{0}\binom{5}{0}\binom{14}{8}^{-1} = 0.0007$$

when $Z = 3$:

$$P\{Z = 2k + 1\} = \left\{\binom{7}{1}\binom{5}{0} + \binom{7}{0}\binom{5}{1}\right\}\binom{14}{8}^{-1} = 0.004$$

and when $Z = 4$:

$$P\{Z = 2k\} = 2\binom{7}{1}\binom{5}{1}\binom{14}{8}^{-1} = 0.0233$$

The sum of these probabilities is 0.028, which is significant at the 5% level.

Hypothetical recombinant: G4/G5 (Supplementary Fig. 2D)

Because G4/G5 is located in the corner opposite the outgroup and G3 (E20) and G9 are located in the two corners of another diagonal, G4/G5 is a hypothetical recombinant and G3 (E20) and G9 are the hypothetical parents. Side 1 comprises 12 PISs (colored red), whereas side 2 comprises 8 PISs (colored blue). The order of PISs is as follows (PISs derived from side 2 are shown in bold typeface):

75, 76, 77, 93, 95, 226, 445, 489, **660, 745, 987, 988, 1001, 1039, 1217, 1218,** 1244, 1245, 1292, 1293

The number of runs (*Z*) is 3 (75-489, 660-1218 and 1244-1293), the number of PISs originating from side 1 (α) is 12 and the number of PISs originating from side 2 (β) is 8. The total number of PISs (n) is 20. Therefore, the sum of the probability of the number of runs (Z) was calculated to be 2 and 3. When $Z = 2$:

$$P\{Z = 2k\} = 2\binom{11}{0}\binom{7}{0}\binom{20}{10}^{-1} = 2.0\times10^{-5}$$

and when $Z = 3$:

$$P\{Z = 2k + 1\} = \left\{\binom{11}{1}\binom{7}{0} + \binom{11}{0}\binom{7}{1}\right\}\binom{20}{12}^{-1} = 0.00014$$

The sum of these probabilities is 0.00016, which is significant at the 0.05% level.

Hypothetical recombinant: G3 (E19 and E22) (Fig. S2E)

Because G3 (E19 and E22) are located in the corner opposite the outgroup and G2/G7 and G3 (E20) are located in the two corners of another diagonal, G3 (E19 and E22) is a hypothetical recombinant and G2/G7 and G3 (E20) are the hypothetical parents. Side 1 comprises 20 PISs (colored red), whereas side 2 comprises 4 PISs (colored blue). The order of PISs is as follows (PISs from side 2 are shown in bold typeface):

199, 218, 445, **459, 460, 473, 474,** 489, 660, 745, 824, 838, 848, 876, 1136, 1137, 1244, 1245, 1292, 1293, 1355, 1356, 1366, 1376

The number of runs (*Z*) is 3 (199-445, 459-474 and 489-1376), the number of PISs derived from side 1 (α) is 20 and the number of PISs derived from side 2 (β) is 4. The total number of PISs (n) is 24. Therefore, the sum of the probability of the number of runs (*Z*) was calculated to be 2 and 3.

When $Z = 2$:

$$\mathrm{P}\{Z = 2k\} = 2\binom{19}{0}\binom{3}{0}\binom{24}{20}^{-1} = 0.0002$$

and when $Z = 3$:

$$\mathrm{P}\{Z = 2k + 1\} = \left\{\binom{19}{1}\binom{3}{0} + \binom{19}{0}\binom{3}{1}\right\}\binom{24}{20}^{-1} = 0.0021$$

The sum of these probabilities is 0.0023, which is significant at the 0.5% level.

Computer simulation of negative and positive control data sets

In order to generate a 'training set' of negative control data and positive control data, we conducted computer simulations that mimic the evolution of four sequences. In this simulation, we questioned only four sequences because the probability of a false positive rate for each reticule that was observed in this study (**Supplementary Fig. 2A to 2E**) should be calculated. For negative and positive controls, we first generated four sequences following the evolutionary model in **Supplementary Fig. 3**, and then calculated the P value of two-sample runs test. All point mutations occurred by following a site-by-site substitution rate, which is calculated from actual *Enterobacter* representative data. The site-by-site substitution rate was calculated as follows:

$$P_n = r_n \times P_{mean}$$

Where P_n is the substitution rate at site n, r_n is the relative substitution rate at site n, and P_{mean} is the overall substitution rate. r_n and P_{mean} were calculated using MEGA 6.0 software (Tamura et al., 2013) based on the Tamura-Nei model (Tamura and Nei, 1993). A discrete Gamma (+G) distribution was used to model the substitution rate differences among the sites (5 categories).

For the calculation of negative control data, four sequences were generated from the ancestor (**Supplementary Fig. 3A**). From the ancestor, mutations L1 and further mutations L2 generated sequence 1, as well as further mutations L3 generated sequence 2. Sequence 3 and the outgroup were generated by mutations L4 and L5 from the ancestor, respectively. The number of mutations L1 to L5 were randomly decided in the range as follows:

L1: 11.3 to 17.7
L2: 13.5 to 19.0
L3: 1.5 to 22.0
L4: 3.2 to 12.3
L5: 22.4 to 42.3

These parameters were calculated from each phylogenetic tree in **Supplementary Fig. 4**. These phylogenetic trees are the trees from sequences containing five recombination events (**Supplementary Fig. 2**, see also **Table 2**). The recombination event in **Supplementary Fig. 2E** was eliminated because one parent sequence clustered into the cluster of recombinant sequences. In order to calculate the maximum probability of a false positive rate, we used the longest branches from the phylogenetic tree in **Supplementary Fig. 4** for L1 to L5. Furthermore, in order to calculate the maximum false positive rate, we considered all multiple substitutions as parallel mutations in this simulation.

Using the above parameter and model, we carried out two-sample runs test for the site distribution, which consists of the sites that came from L1 against the site that occurred in a parallel mutation. We did 100 000 times resampling. In the result, we fixed the false positive rate of two-sample runs test for *Enterobacter* data as 2.3% when we defined 0.05 P value of two-sample runs test as the threshold.

However, 28.35% of recombination events were detected using 5% criteria by two-sample runs test from the simulation with recombination. In this simulation, four sequences were generated by following the evolutionary history in **Supplementary Fig. 3B**. The number of mutations L1 and L2 were randomly decided in the range as follows:

L1: 9.8 to 15.5
L2: 15.3 to 28.5

These parameters were calculated from each phylogenetic tree in **Supplementary Fig. 5**. These phylogenetic trees were reconstructed with only the two parents and the outgroup sequence for each of the five recombination events (**Supplementary Fig. 2**, see also **Table 2**). The recombination event in **Supplementary Fig. 2E** was eliminated because one of the parent sequences clustered into the cluster of recombinant sequences. In this simulation, we decided on two recombination breaking points because all recombination events crossover at least two times. The two recombination breaking points were randomly selected from the following:

Site at 341
Site at 447
Site at 452
Site at 481
Site at 574
Site at 730
Site at 1231
Site at 1286

The recombination breaking points were the mid-point between the end PISs from side 1 and side 2. For example, site 730 and 1286 were selected from the distribution of the PISs of the recombination event in **Supplementary Fig. 2A**. The arrangement of PISs on sides 1 and 2, which consist the 2 side of reticule in **Supplementary Fig. 2A,** is as follows (the PISs from side 2 are shown in bold typeface):

75, 76, 77, 93, 95, 226, 459, 460, 473, 474, **987, 988, 1001, 1039, 1217, 1218**, 1355, 1367

The end of the PISs from side 1 are 474 and 1355, whereas the end of the PISs from side 2 are 987 and 1218.

Using the above parameter and model, we carried out a two-sample runs test for the site distribution from the 2 side of the reticule of the phylogenetic network. When recombination occurs between parent 1 and parent 2 whose recombination breaking point is i and j (**Supplementary Fig. 6A**), the reticule of the phylogenetic network should consist of a side whose PISs are $X^{i\text{-}j}$ and another side whose PISs are $Y^{1\text{-}i,\, j\text{-}n}$ (**Supplementary Fig. 6B**). X and Y indicate the accumulated substitutions from the ancestor to parent 1 and parent 2, respectively. i, j, and n indicate the first recombination breaking point, second recombination breaking point, and the length of the sequence, respectively. Thus, $X^{i\text{-}j}$ indicates the substitution X in the range from site i to j. We did 100 000 times resampling. In the result, 28.35% of recombination events were detected using 5% criteria. The 2 simulations indicate that the false positive rate is less than 3% by 5% criteria and that criteria have enough detecting power for the recombination of the *Enterobacter* 16S rRNA gene.

Supplementary figure legends

Supplementary Figure 1 | Phylogeny of 16S rRNA Gene Copies in *Enterobacter* Genomes. Each phylogeny was reconstructed using the neighbor-joining method (Saitou and Nei, 1987) with the Kimura two-parameter distance (Kimura, 1980) for all sets of 16S rRNA genes from (*A*) G1, (*B*) G2, (*C*) G3, (*D*) G4, (*E*) G5, (*F*) G8 and (*G*) G10. Representative sequences of each genome are indicated by black circles.

Supplementary Figure 2 | Parsimonious Phylogenetic Network for Individual Groups. In each reticule, side 1 is colored red and side 2 in light blue. The numbers along each side are the nucleotide positions responsible for those sides. The numbers prefixed with "S" are the numbers of singletons. The highlighted network consisted of (*A*) G9, G6/G8 and G2/G7; (*B*) G1, G5 and G6/G8; (*C*) G2/G7, G3 (E20) and G9; (*D*) G3 (E20), G4, G5 and G9, and (*E*) G3 and G2/G7.

Supplementary Figure 3 | Explanation for Two Evolutionary Simulation Models. (A) Evolutionary model of four sequences without considering recombination. From the "Ancestor," the mutation L1 and the additional mutation L2 and L3 generate "Sequence 1" and "Sequence 2," respectively. "Sequence 3" and the "Outgroup" were separated from the "Ancestor" by L4 and L5 mutations, respectively. (B) Evolutionary model for four sequences ("Ancestor," "Sequence 1," "Sequence 2," and "Recombinant") when considering recombination. "Sequence 1" and "Sequence 2" are generated by the accumulation of point mutations L1 and L2 to the "Ancestor," respectively. Recombination between "Sequence 1" and "Sequence 2" generates the "Recombinant".

Supplementary Figure 4 | Phylogenetic Trees among the Sequences of Each Recombination Event without Considering Recombination. The sequences of each tree are: (A) of **Supplementary Fig. 2A**, (B) of **Supplementary Fig. 2B**, (C) of **Supplementary Fig. 2C,** and (D) of **Supplementary Fig. 2E**. The phylogenetic tree was reconstructed using the maximum parsimony method and the Subtree-Pruning-Regrafting (SPR) algorithm (Nei and Kumar, 2000).

Supplementary Figure 5 | Phylogenetic Trees among the Sequences of Each

Recombination Event Considering Recombination. The sequences of each tree are: (A) of **Supplementary Fig. 2A**, (B) of **Supplementary Fig. 2B**, (C) of **Supplementary Fig. 2C** and (D) of **Supplementary Fig. 2E.** All recombinant sequences were removed from the analysis. The phylogenetic tree was reconstructed using the maximum parsimony method and the Subtree-Pruning-Regrafting (SPR) algorithm (Nei and Kumar, 2000).

Supplementary Figure 6 | Explanation for the PISs Distribution on the 2 Side of the Reticule in the Phylogenetic Network. (A) Evolutionary model with recombination. (B) Phylogenetic network among the four sequences ("Outgroup," "Parent 1," "Parent 2," and "Recombinant"). In this model, we assume that mutation $X^{1\text{-}n}$ and $Y^{1\text{-}n}$ to the ancestor sequence (outgroup) generates "Parent 1" and "Parent 2," respectively, and the recombination between "Parent 1" and "Parent 2" generates the "Recombinant" (the recombination breaking point is i and j). The phylogenetic network for this evolutionary model is shown in (B). The superscript letters indicate the region on the sequence. For example, $X^{i\text{-}j}$ indicates the substitution sites in the "Parent 1" lineage at the region from site i to j.

References

1. Ezawa K, Oota S, Saitou N (2006) Genome-wide search of gene conversions in duplicated genes of mouse and rat. *Mol Biol Evol* 23(5):927–940.
2. Kimura M. (1980) A simple method for estimating evolutionary rate of base substitutions through comparative studies of nucleotide sequences. *J Mol Evol* 16(2):111–120.
3. Nei, M. Kumar, S. (2000). Molecular Evolution and Phylogenetics. Oxford University Press, New York.
4. Saitou N, Nei M (1987) The neighbor-joining method: A new method for reconstructing phylogenetic trees. *Mol Biol Evol* 4(4):406–425.
5. Takahata N (1994) Comments on the detection of reciprocal recombination or gene conversion. *Immunogenetics* 39(2):146–149.
