# Supplementary material for: Phylogenetic Network Analysis Revealed the Occurrence of Horizontal Gene Transfer of 16S rRNA in the Genus Enterobacter
Source: Front Microbiol. 2017 Nov 16;8:2225. doi: 10.3389/fmicb.2017.02225 (PMC5688380; doi:10.3389/fmicb.2017.02225)
Supplement: Supplementary file 4 [file Data_Sheet_2.PDF]

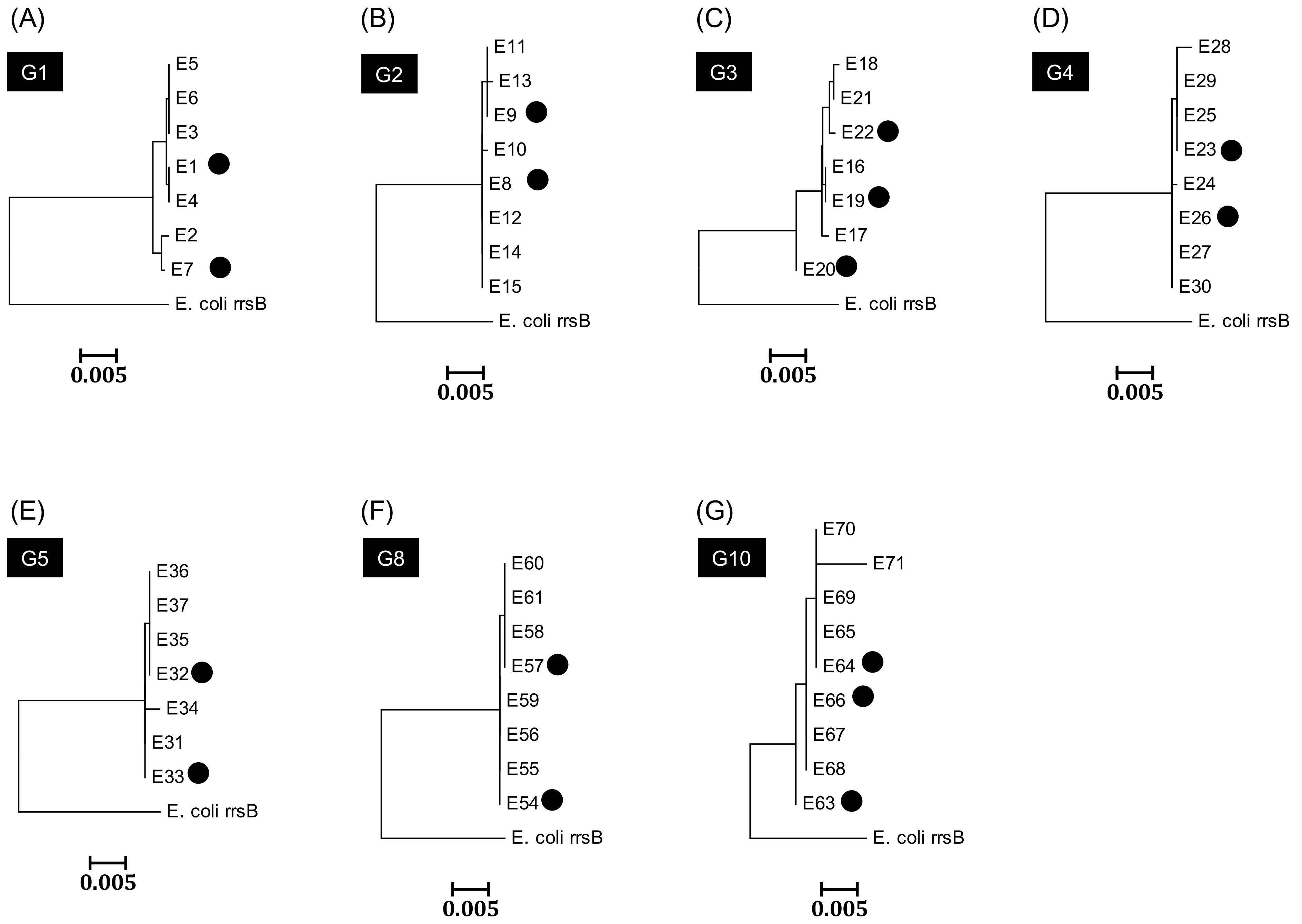
(A)
G1
E5
E6
E3
E1
E4
E2
E7
E. coli rrsB
0.005
(B)
G2
E11
E13
E9
E10
E8
E12
E14
E15
E. coli rrsB
0.005
(C)
G3
E18
E21
E22
E16
E19
E17
E20
E. coli rrsB
0.005
(D)
G4
E28
E29
E25
E23
E24
E26
E27
E30
E. coli rrsB
0.005
(E)
G5
E36
E37
E35
E32
E34
E31
E33
E. coli rrsB
0.005
(F)
G8
E60
E61
E58
E57
E59
E56
E55
E54
E. coli rrsB
0.005
(G)
G10
E70
E71
E69
E65
E64
E66
E67
E68
E63
E. coli rrsB
0.005

Figure S1

(A)

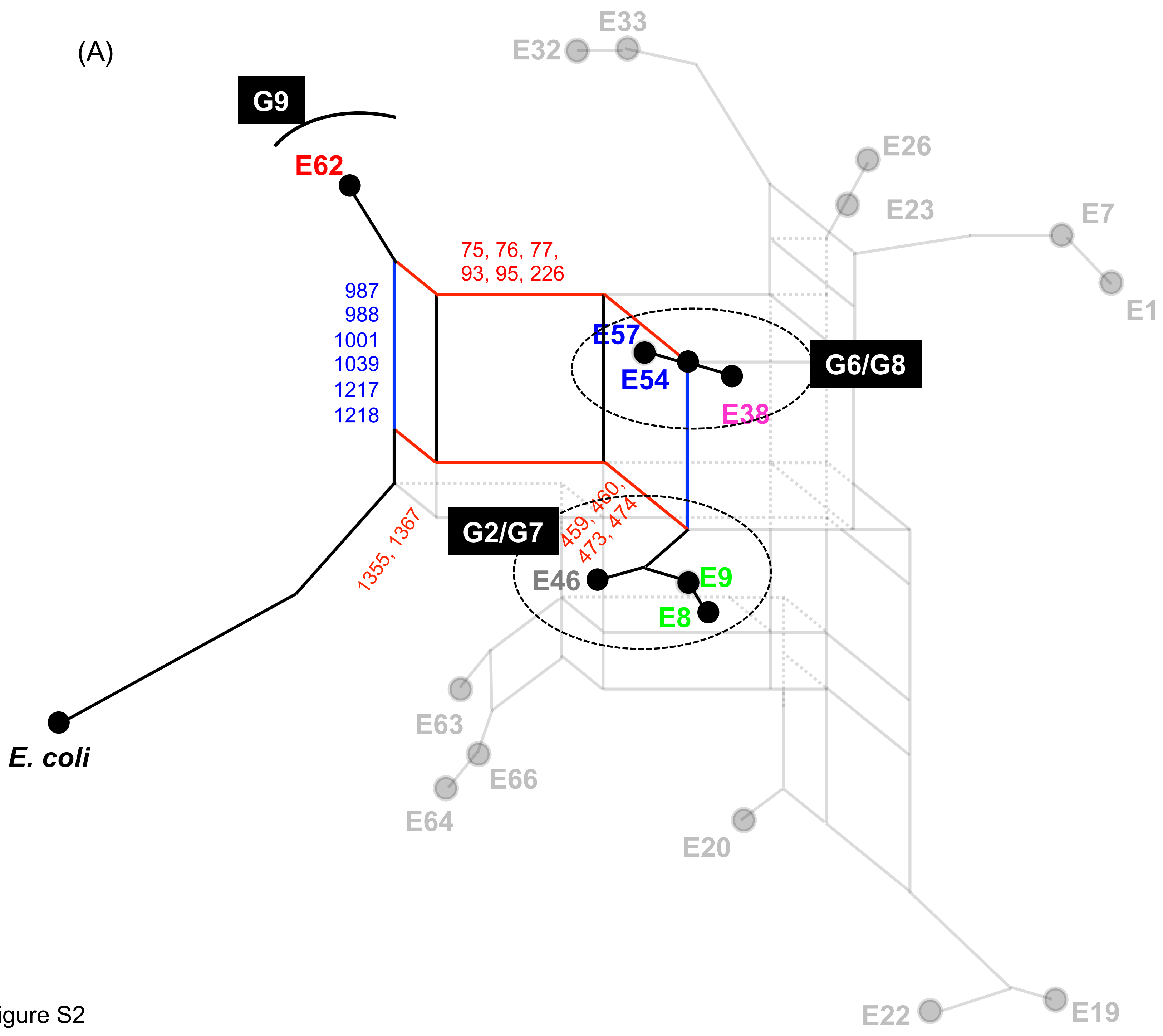
E33
E32
G9
E26
E62
E23
E7
75, 76, 77,
93, 95, 226
987
988
1001
1039
1217
1218
E1
E57
G6/G8
E54
E38
459, 460,
473, 474
1355, 1367
G2/G7
E46
E9
E8
E63
E. coli
E66
E64
E20
E22
E19

Figure S2

(B)

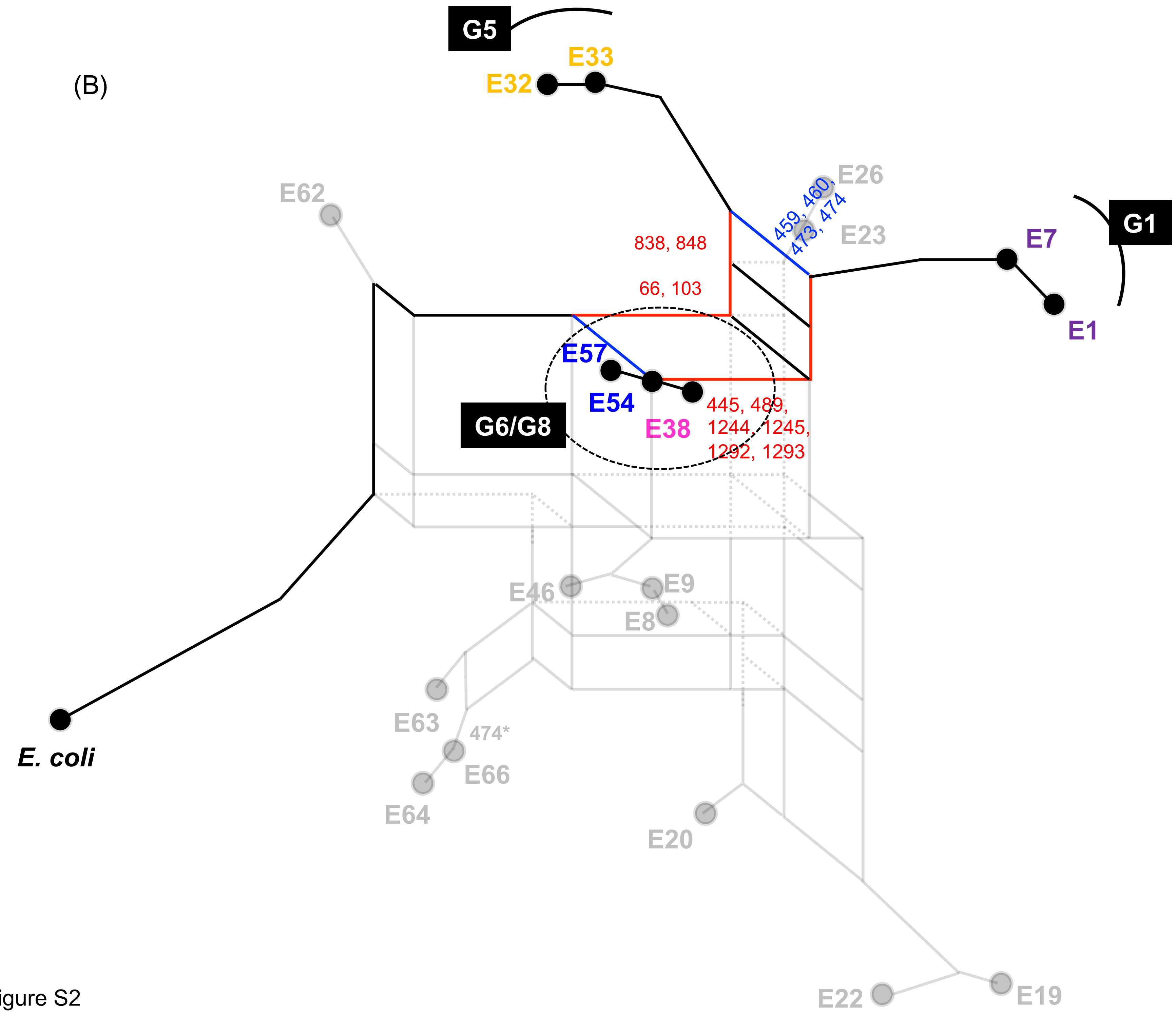
G5
E33
E32
E26
459, 460,
473, 474
E23
E62
838, 848
G1
E7
66, 103
E1
E57
E54
445, 489,
1244, 1245,
1292, 1293
G6/G8
E38
E46
E9
E8
E63
474*
E66
E64
E20
E. coli
E22
E19

Figure S2

(C)

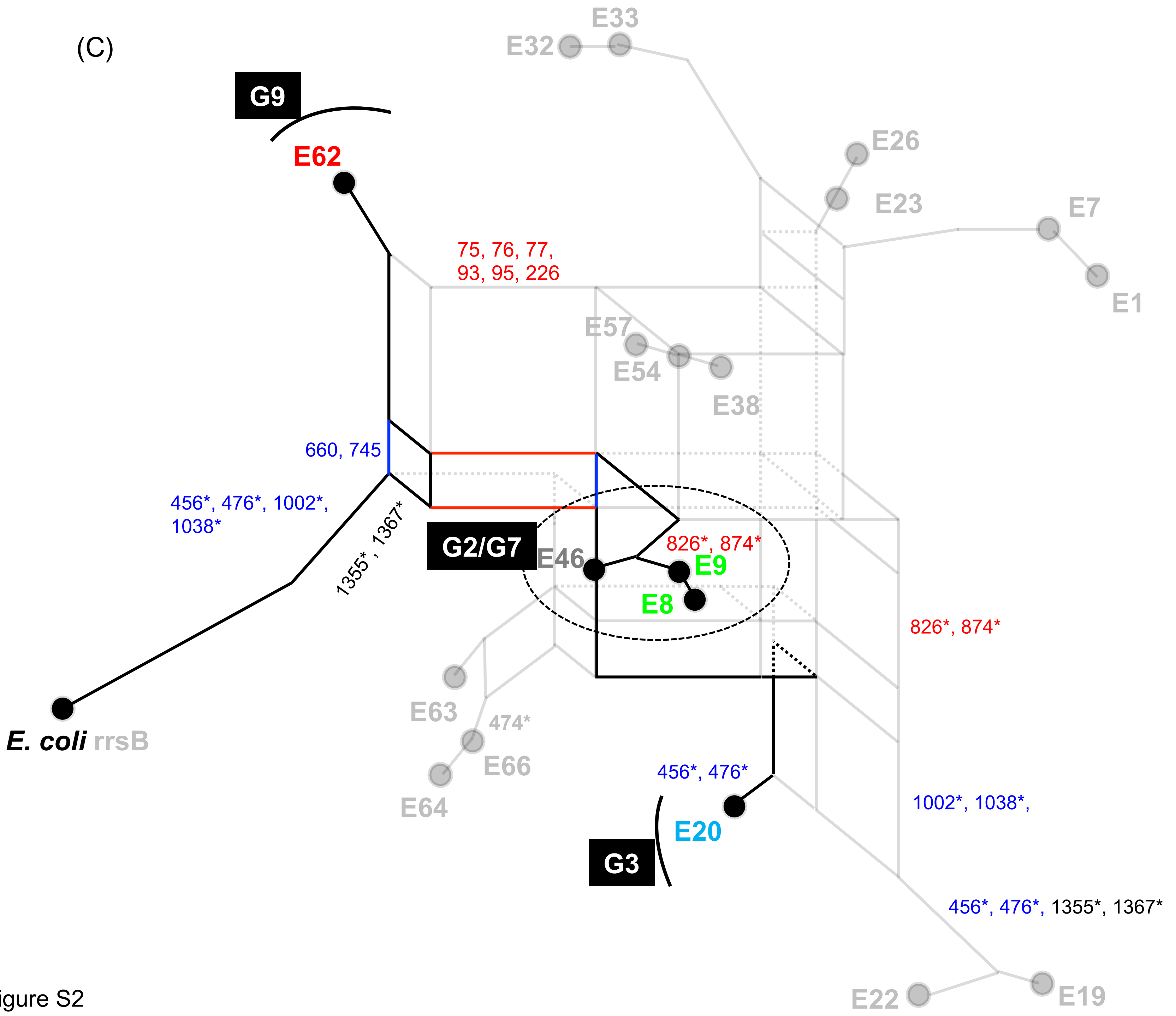
E33
E32
G9
E26
E62
E23
E7
75, 76, 77,
93, 95, 226
E1
E57
E54
E38
660, 745
456*, 476*, 1002*,
1038*
1355*, 1367*
G2/G7
E46
826*, 874*
E9
E8
826*, 874*
E63
474*
E. coli rrsB
E66
456*, 476*
1002*, 1038*,
E64
E20
G3
456*, 476*, 1355*, 1367*
E22
E19

Figure S2

(D)

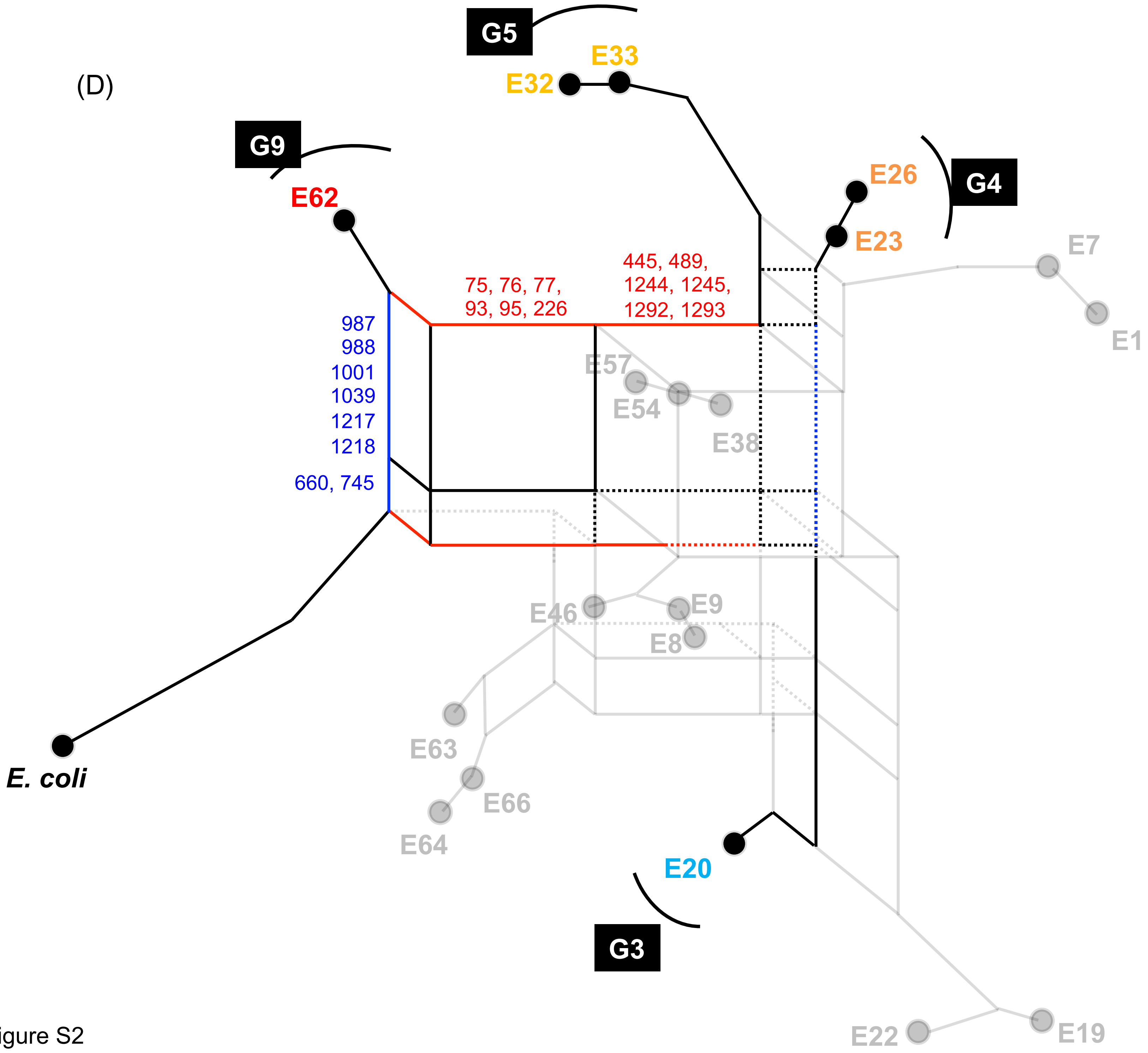
G5
E33
E32
G9
E62
E26
G4
E23
E7
445, 489,
1244, 1245,
1292, 1293
75, 76, 77,
93, 95, 226
E1
987
988
1001
1039
1217
1218
E57
E54
E38
660, 745
E46
E9
E8
E. coli
E63
E66
E64
E20
G3
E22
E19

Figure S2

(E)

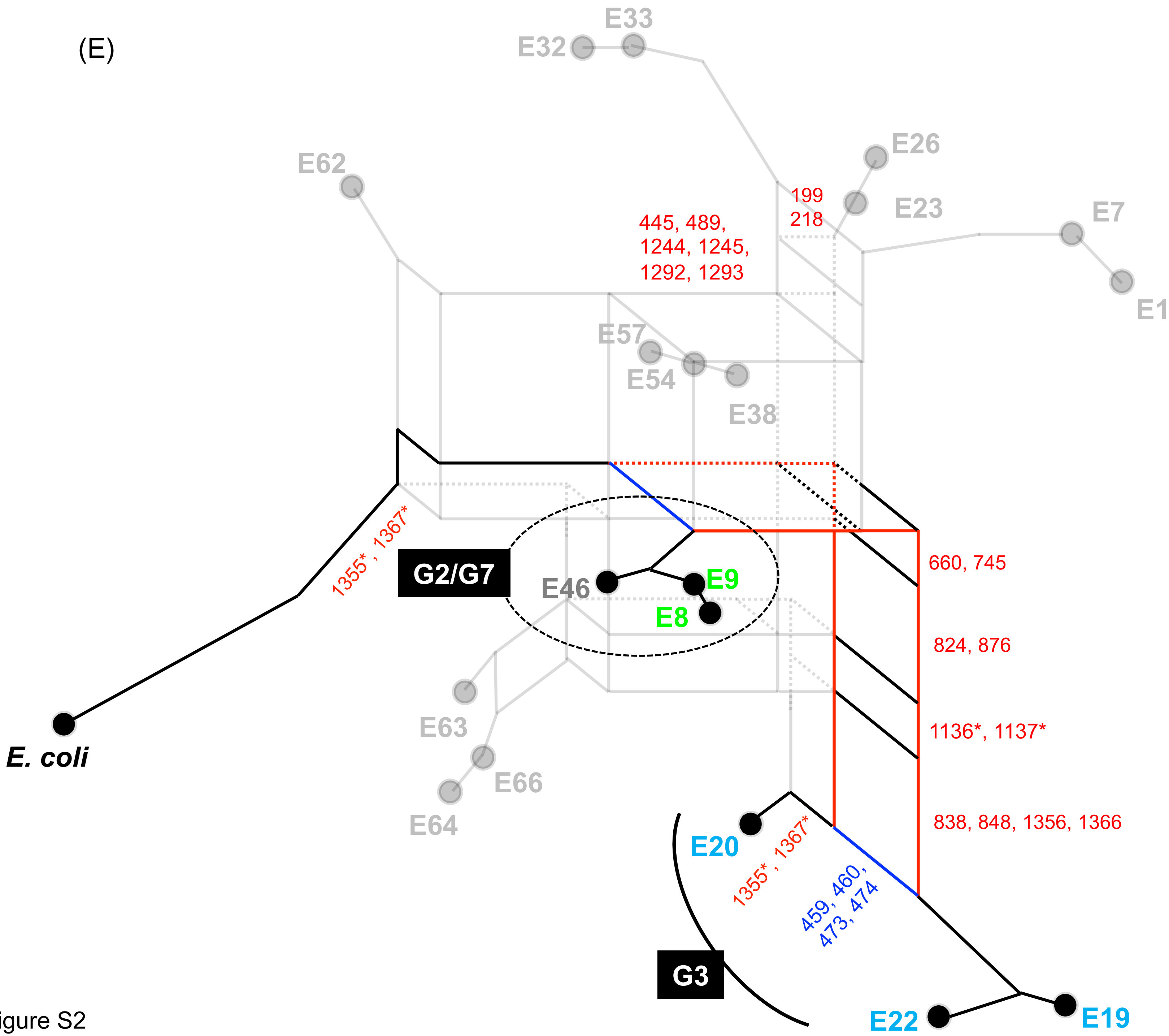
E33
E32
E26
E62
199
218
E23
E7
445, 489,
1244, 1245,
1292, 1293
E1
E57
E54
E38
1355*, 1367*
G2/G7
E46
E9
E8
660, 745
824, 876
1136*, 1137*
E. coli
E63
E66
E64
838, 848, 1356, 1366
E20
1355*, 1367*
459, 460,
473, 474
G3
E22
E19

Figure S2

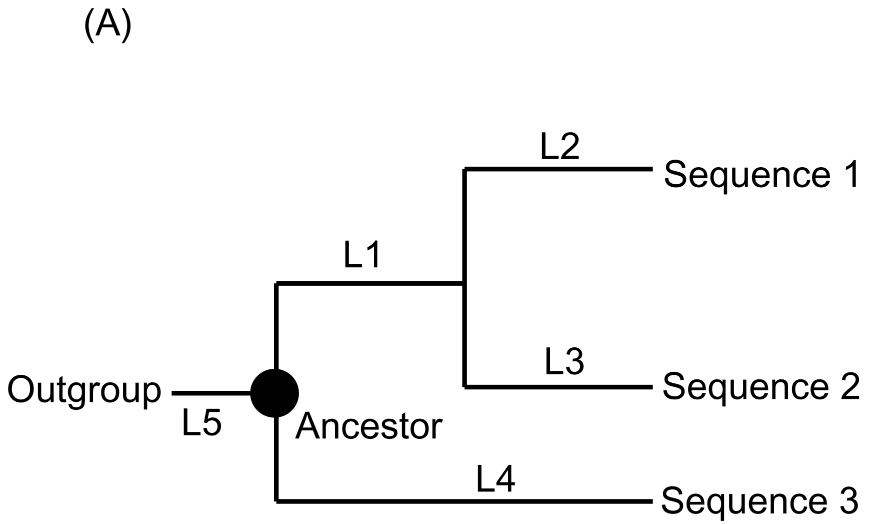
(A)
L2
Sequence 1
L1
L3
Outgroup
Sequence 2
L5
Ancestor
L4
Sequence 3

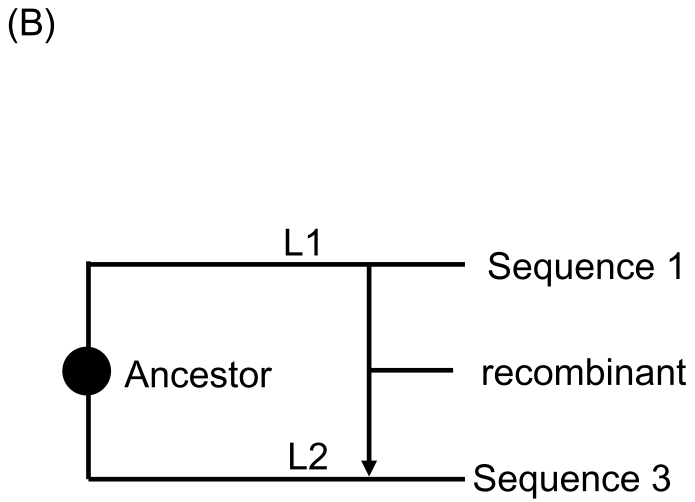
(B)
L1
Sequence 1
Ancestor
recombinant
L2
Sequence 3

Figure S3

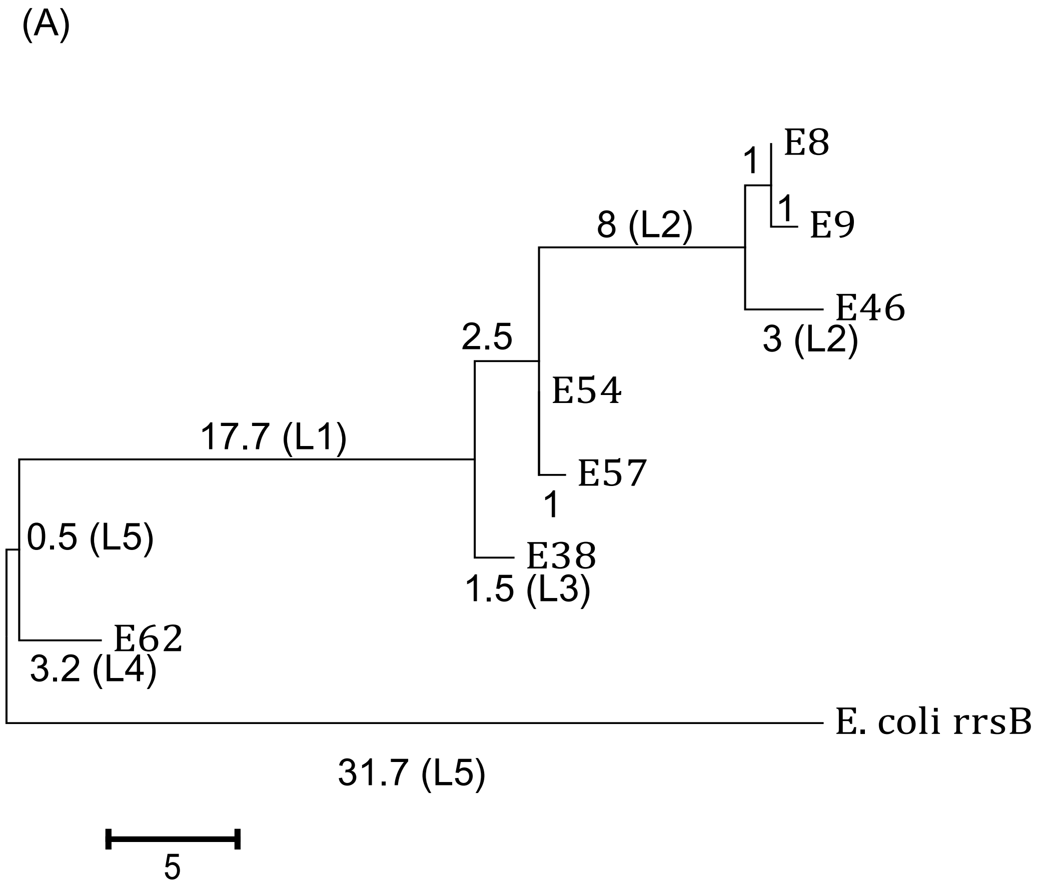
(A)
E8
1
1
E9
8 (L2)
E46
3 (L2)
2.5
E54
17.7 (L1)
E57
1
0.5 (L5)
E38
1.5 (L3)
E62
3.2 (L4)
E. coli rrsB
31.7 (L5)
5

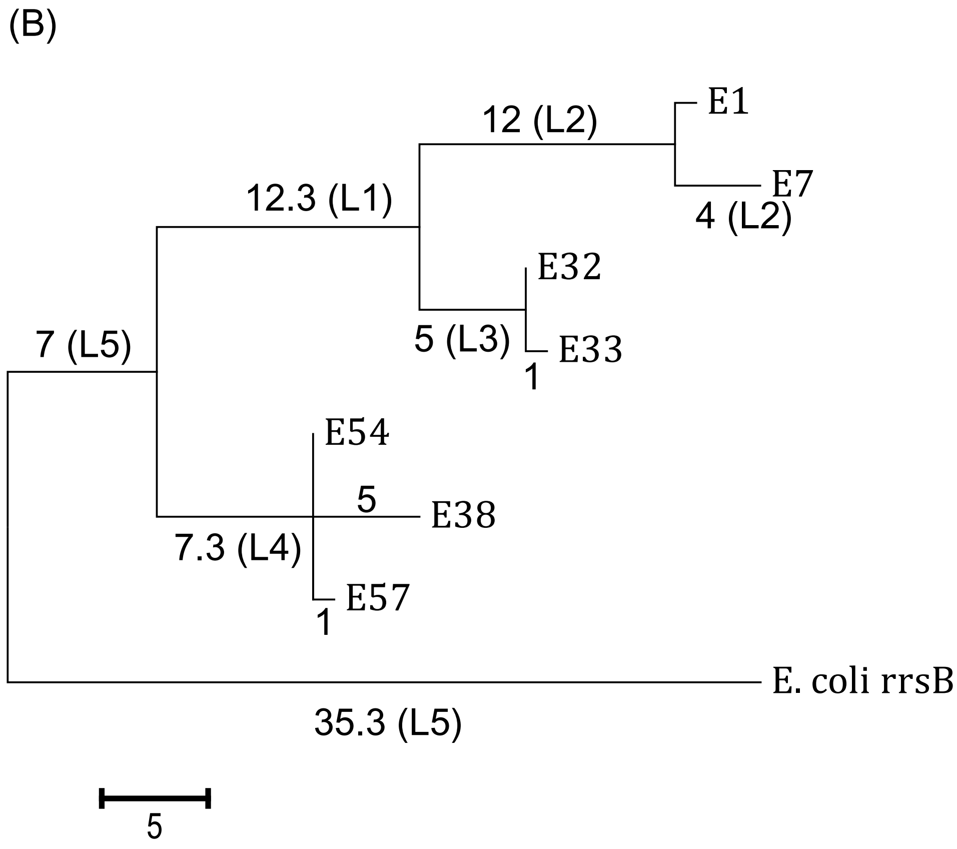
(B)
E1
12 (L2)
E7
12.3 (L1)
4 (L2)
E32
7 (L5)
5 (L3)
E33
1
E54
5
E38
7.3 (L4)
E57
1
E. coli rrsB
35.3 (L5)
5

Figure S4-1

(C) (D)

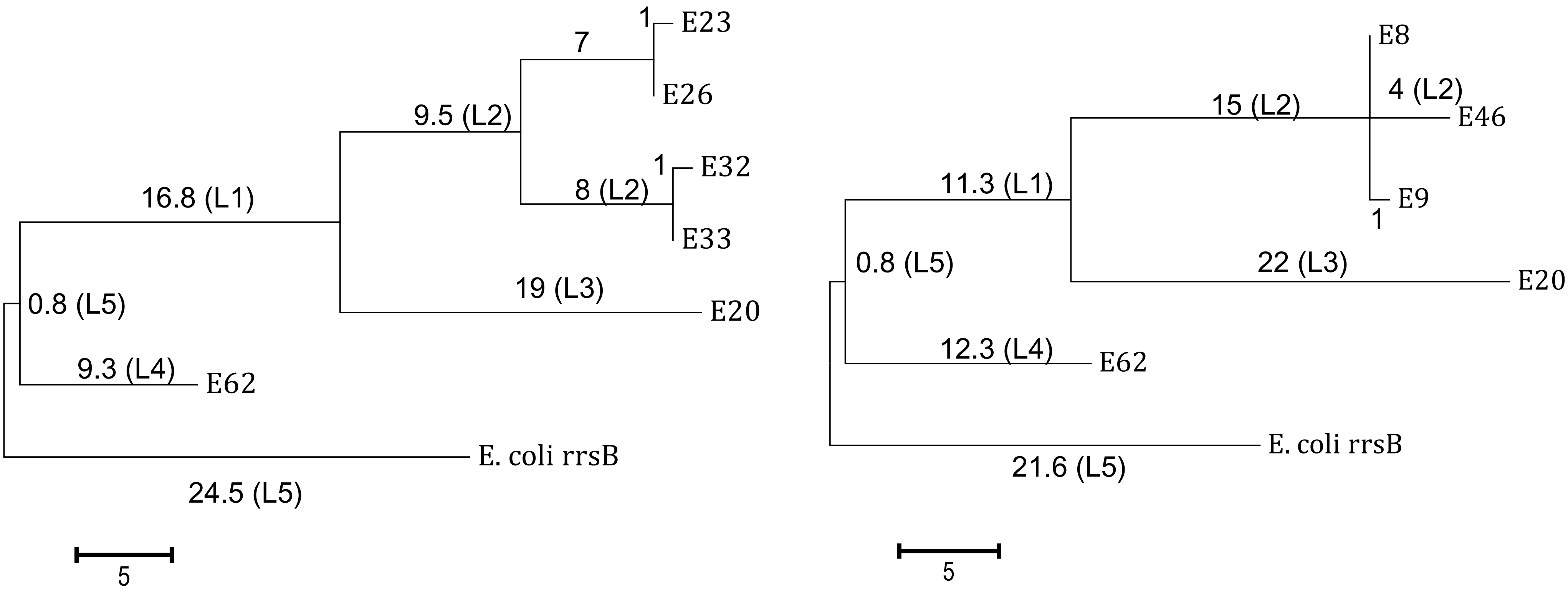

Figure S4-2

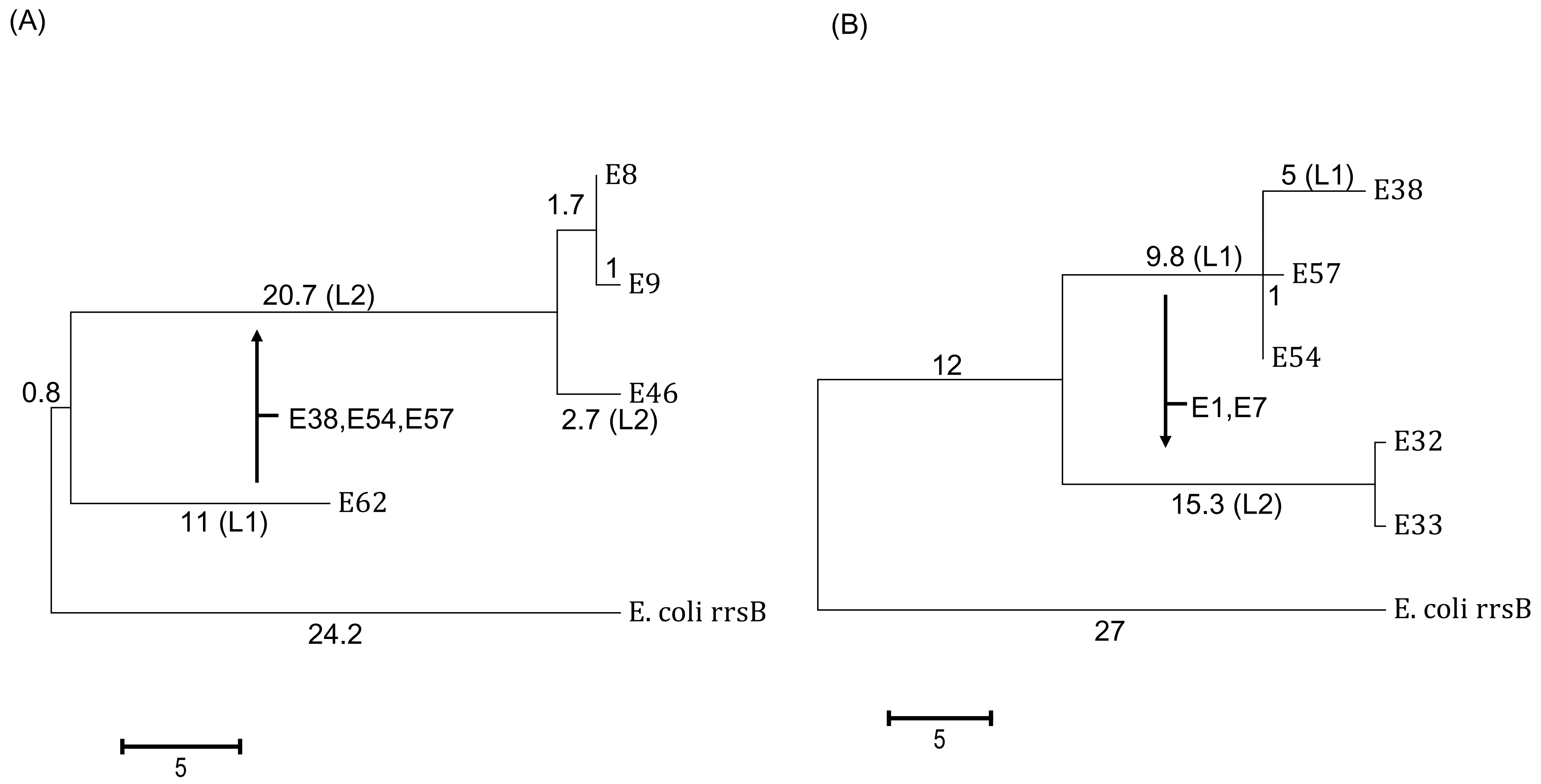
(A)
E8
1.7
1
E9
20.7 (L2)
0.8
E38,E54,E57
E46
2.7 (L2)
E62
11 (L1)
E. coli rrsB
24.2
5
(B)
5 (L1)
E38
9.8 (L1)
E57
1
E54
12
E1,E7
E32
15.3 (L2)
E33
E. coli rrsB
27
5

Figure S5-1

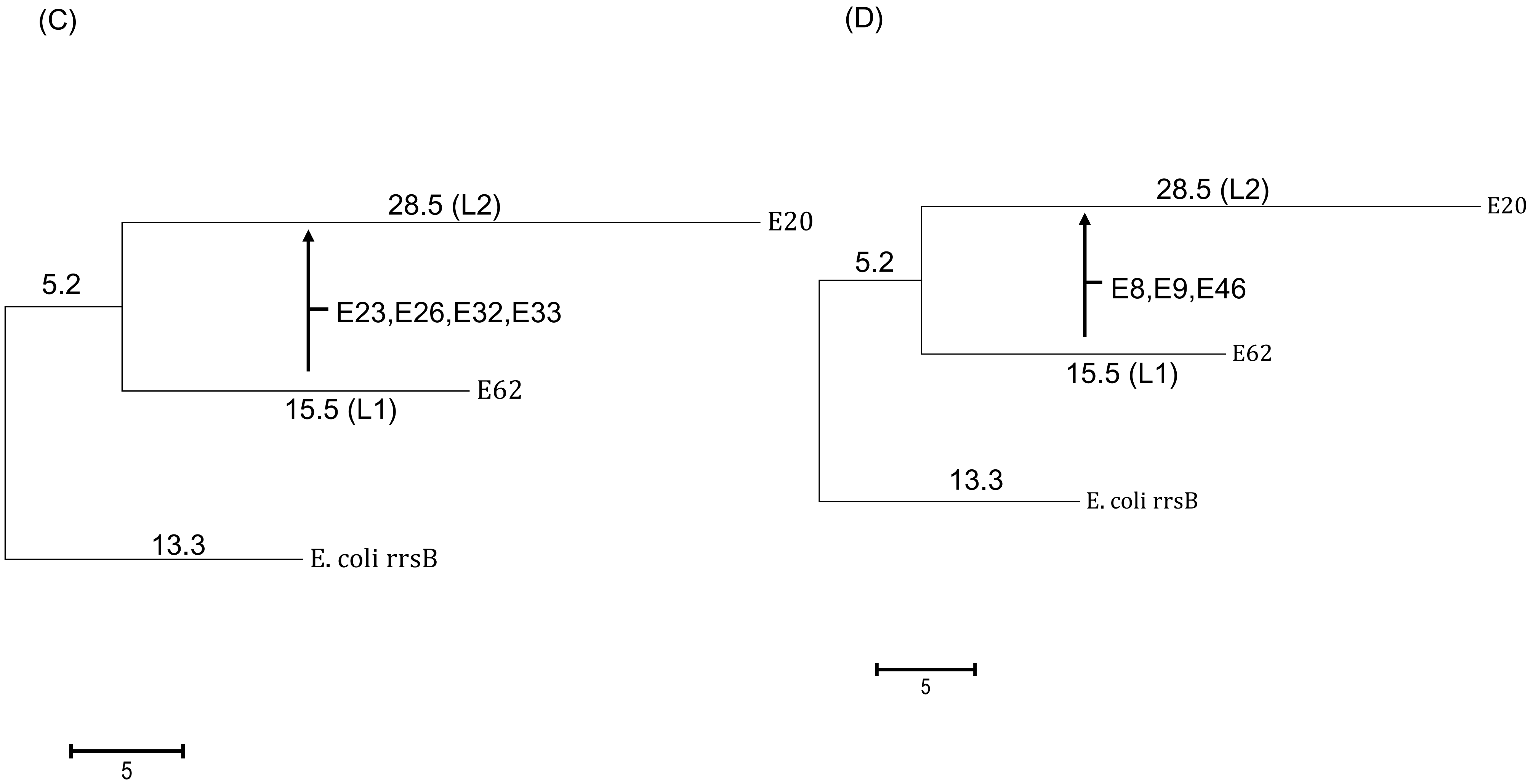
(C)
28.5 (L2)
E20
5.2
E23,E26,E32,E33
E62
15.5 (L1)
13.3
E. coli rrsB
5
(D)
28.5 (L2)
E20
5.2
E8,E9,E46
E62
15.5 (L1)
13.3
E. coli rrsB
5

Figure S5-2

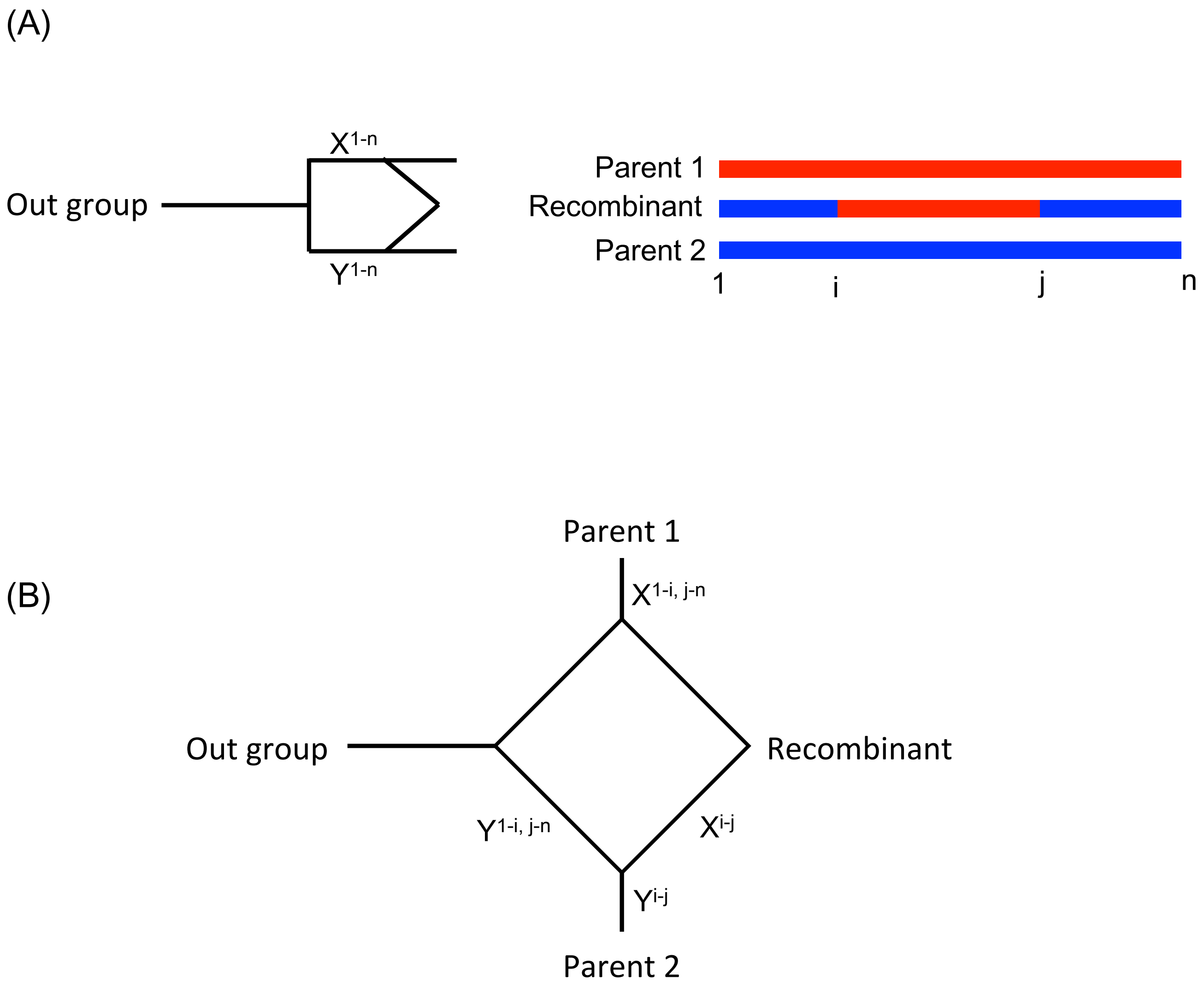

(A)
Out group
X1-n
Y1-n
Parent 1
Recombinant
Parent 2
1
i
j
n
(B)
Parent 1
X1-i, j-n
Out group
Recombinant
Y1-i, j-n
Xi-j
Yi-j
Parent 2

Figure S6
